# FBXO39 promotes LDHA-mediated aerobic glycolysis and colorectal cancer progression by p53 degradation

**DOI:** 10.1186/s12967-026-08056-7

**Published:** 2026-04-01

**Authors:** Jipeng Liu, Taifu Xiao, Jun Huang

**Affiliations:** 1 Department of Gastrointestinal Surgery, The Second Affiliated Hospital, Jiangxi Medical College, Nanchang University, Nanchang, Jiangxi Province 330006 PR China; 2 Precision Oncology Medicine Center, The Second Affiliated Hospital, Jiangxi Medical College, Nanchang University, Nanchang, Jiangxi Province 330006 PR China

**Keywords:** FBXO39, p53, LDHA, Aerobic glycolysis, Colorectal cancer

## Abstract

**Background:**

Colorectal cancer (CRC) is one of the leading causes of cancer-related deaths worldwide. Ubiquitination modification is extensively involved in various biological processes, including aerobic glycolysis and tumor development. TP53 mutations are present in nearly half of CRC patients, while in p53-wild type CRC, effective therapeutic targets remain relatively limited.

**Methods:**

We employed bioinformatics methods to systematically analyze the expression and prognostic value of E3 ubiquitin ligase FBXO family members in CRC, with a focus on FBXO39. Through in vitro experiments such as CCK-8 assay, EdU cell proliferation assay, colony formation assay, glucose metabolite measurement, oxygen consumption rate (OCR) and extracellular acidification rate (ECAR) analyses, as well as in vivo experiments including cell-derived xenograft models, we elucidated the biological functions of FBXO39 in CRC. Furthermore, we explored its molecular mechanisms through molecular docking, co-immunoprecipitation (Co-IP), and ubiquitination assays.

**Results:**

FBXO39 expression was significantly higher in CRC tissues than in adjacent normal tissues, and its high expression was associated with poor patient prognosis. Functional experiments demonstrated that FBXO39 significantly promoted CRC cell proliferation both in vitro and in vivo. Mechanistically, FBXO39 directly bound to the p53 protein and promoted its ubiquitination-mediated degradation in p53-wild type CRC cells. Further investigations revealed that FBXO39 overexpression enhanced the aerobic glycolysis capacity of CRC cells, while its knockdown inhibited this process. Mechanistically, FBXO39 enhanced aerobic glycolysis and promoted tumor cell proliferation by upregulating lactate dehydrogenase A (LDHA) expression in a p53-dependent manner.

**Conclusion:**

This study reveals a novel mechanism for p53 protein downregulation in p53-wild type CRC, elucidates the role of FBXO39 in regulating aerobic glycolysis through the p53/LDHA axis to promote tumor progression, and provides new insights into the function of FBXO39 in metabolic reprogramming in CRC.

**Supplementary information:**

The online version contains supplementary material available at 10.1186/s12967-026-08056-7.

## Introduction

Colorectal cancer (CRC) is one of the leading causes of global cancer-related morbidity and mortality [1]. CRC patients diagnosed early and undergoing radical resection often achieve favorable clinical outcomes, with a five-year survival rate exceeding 90% [2]. However, due to the lack of specific early symptoms, many patients are diagnosed at an advanced or even late stage [3]. Treatment options for advanced CRC are limited; surgical resection is often not feasible, and current systemic therapies, such as chemotherapy, targeted therapy, and immunotherapy, are generally suboptimal, resulting in a five-year survival rate of less than 15% and a high propensity for recurrence [4]. Therefore, identifying reliable prognostic biomarkers could better predict patient survival outcomes.

Metabolic reprogramming is a fundamental hallmark of malignant progression in cancer cells [5]. Aerobic glycolysis, known as the Warburg effect, plays a critical role in the progression of various malignancies, including CRC [6–8]. P53, a central transcription factor, plays a pivotal role in maintaining genomic integrity, regulating cell cycle progression, and modulating metabolic pathways [9]. Although the p53 tumor suppressor pathway exerts anti-cancer functions in various cancers, this pathway is often inactivated in many tumors [10, 11]. For instance, in CRC, TP53 gene mutations occur in approximately half of all patients [12, 13]. Mutant p53 loses its wild-type function, failing to induce cell cycle arrest and apoptosis, thereby allowing cells with DNA damage to proliferate continuously, accumulate more mutations, and ultimately drive malignant tumor progression [14]. Notably, even in CRC with wild-type TP53, p53 protein expression is often low, the reasons and specific mechanisms for which are not yet fully understood.

Ubiquitination, as a crucial post-translational modification, involves a cascade of enzymes including E1 ubiquitin-activating enzymes, E2 ubiquitin-conjugating enzymes, and E3 ubiquitin ligases [15, 16]. This process, by specifically targeting proteins and labeling them with specific polyubiquitin chains, guides proteasome-dependent degradation, regulates protein stability, and is extensively involved in processes such as cell cycle control, metabolic reprogramming, and tumor development. The FBXO family is an important subfamily of F-BOX proteins within the E3 ubiquitin ligases, playing a key role in the ubiquitination process [17, 18]. Several FBXO members are involved in CRC progression by regulating the ubiquitination of specific substrates. For example, FBXO8 and FBXO32 exhibit downregulated expression in CRC tissues and act as tumor suppressors [19, 20]. Conversely, FBXO5, FBXO22, and FBXO44 show upregulated expression in CRC tissues and promote tumor progression [21–23]. Multiple bioinformatics studies have indicated associations between FBXO39 and tumors such as breast cancer, cervical squamous cell carcinoma, and osteosarcoma, suggesting its potential prognostic value [24, 25]. However, the specific biological role of FBXO39 in CRC and its underlying molecular mechanisms remain unclear.

In this study, we first established the biological function of FBXO39 in promoting CRC progression. Furthermore, we discovered that FBXO39 promotes the ubiquitination and degradation of the p53 protein. Subsequently, we confirmed that FBXO39, by degrading p53, relieves the inhibitory effect of p53 on lactate dehydrogenase A (LDHA), ultimately driving aerobic glycolysis and tumor cell growth through the upregulation of LDHA.

## Materials and methods

### Bioinformatics analysis

The transcriptomic profiles and comprehensive clinical phenotype data of COAD patients were obtained from the UCSC Xena platform, with progression-free interval (PFI) selected as the primary survival endpoint. All statistical analyses and visualizations were performed using R software (version 4.5.2). Survival analyses, including univariate and multivariate Cox proportional hazards regression, were conducted using the survival and survminer packages, employing a complete-case analysis strategy to handle missing clinical covariates. The proportional hazards (PH) assumption was verified using Log-Log plots.Single-cell RNA sequencing data (dataset GSE178341) were re-analyzed to characterize the cellular distribution of FBXO39. We performed cell type annotation, dimensionality reduction (UMAP), and lineage tracing (Sankey diagrams) to validate the epithelial origin of FBXO39-expressing cells.The protein-protein interaction between FBXO39 and P53 was modeled using the HDOCK server and visualized with PyMOL; the binding affinity was quantitatively predicted via the PRODIGY web server. For transcriptional regulation analysis, the binding of P53 to the LDHA promoter was predicted based on the position weight matrix (PWM) from the JASPAR database. The identification of high-confidence binding sites was performed using a custom Python script (utilizing the NumPy library), and their significance was assessed through Monte Carlo simulations.

### Clinical sample analysis

The CRC specimens used in this study were collected from patients at the Second Affiliated Hospital of Nanchang University. These patients were postoperatively pathologically diagnosed with CRC and had not received neoadjuvant therapy. This cohort included 30 CRC patients, from whom matched tumor and adjacent normal tissue specimens were acquired. All human sample collection procedures were performed with written informed consent from the patients. The study was approved by the Clinical Research Ethics Committee of the Second Affiliated Hospital of Nanchang University ([2019] No. (053)).

### Cell lines and culture

The human CRC cell lines RKO and HCT116 were acquired from BOSTER Biological Technology Co., Ltd. (Wuhan, China). All cell lines in this study were cultured in a sterile, humidified incubator at 37 °C using RPMI-1640 medium (Solarbio, China), supplemented with antibiotics and 10% fetal bovine serum (FBS; Excell, Uruguay).

### Western blotting

The following primary antibodies were used in Western Blotting experiments: anti-FBXO39 (1:1000; Thermo-Fisher, USA), anti-p53 (1:2000; Proteintech, China), anti-LDHA (1:1000; Proteintech, China), anti-NEDD8-Culin-1 (1:2000; Proteintech, China), anti-ubiquitin (1:1000; Proteintech, China), and anti-tubulin (1:10000; Proteintech, China). After electrophoresis and transfer, PVDF membranes were incubated with primary antibodies on a shaker at 4 °C overnight. Subsequently, the membranes were washed three times with TBST (Servicebio, China) and probed with anti-rabbit or anti-mouse secondary antibodies (1:5000; Proteintech, China) for 1 hour. After three TBST washes, bands were visualized using an enhanced chemiluminescence (ECL) system. Finally, images were captured and grayscale quantification was performed using ImageJ software (version 1.51).

### Lentiviral transfection and stable cell line construction

Lentiviruses for FBXO39 knockdown and overexpression were synthesized by Hanbio Biotechnology Co., Ltd. (Wuhan, China). Stable FBXO39 knockdown or overexpression RKO and HCT116 cell lines were obtained through puromycin selection. qRT-PCR and Western blotting confirmed construction efficiency.

### RNA extraction and qRT-PCR

Total RNA was isolated from cell and tissue samples using an RNA extraction kit (Beyotime, Beijing, China). Following extraction, cDNA was synthesized using a reverse transcription kit (Beyotime). qRT-PCR amplification was carried out using a PCR kit (Beyotime), with GAPDH as the endogenous reference gene. The 2^−ΔΔCt^ method was applied to determine relative gene expression. Data visualization was conducted with Prism software (v10.1.2). The nucleotide sequences of the primers used are listed in Table 1.

**Table 1:** Sequences of the primers used for RT-qPCR

Name	Sequence
FBXO39	Forward (5′-GATGGGCAAACGCCTGGATTA-3′) Reverse (5′-GGAGGGTGCTGGCATTCTCAC-3′) Forward (5′-CAGCACATGACGGAGGTTGT-3′） Reverse (5′-TCATCCAAATACTCCACACGC-3′)
P53	
LDHA	Forward (5‘-ATGGAGACTTGTGTTGGCGG-3’）
	Reverse (5’-TCCTTTTCCGCCTTTGACTG-3’)
GAPDH	Forward 5′-TGACTTCAACAGCGACACCCA-3′）
	Reverse (5′-CACCCTGTTGCTGTAGCCAAA-3′)

### Immunohistochemistry (IHC)

After paraffin embedding of tissue samples, sections were cut to a thickness of 3 micrometers. The sections were incubated with anti-FBXO39 antibody (1:200, Thermo Fisher, USA) at 4 °C overnight. The next day, sections were washed three times with PBST, followed by incubation with an HRP-conjugated secondary antibody. After three PBST washes, DAB staining was performed. Finally, images were captured using a microscope.

### CCK-8 assay

A total of 5.0 × 10^3 cells were seeded into each well of 96-well plates. Cell viability was then determined at designated intervals using the Cell Counting Kit-8 (CCK-8; UElandy, China) as per the manufacturer’s protocol. Measurements of absorbance at a wavelength of 450 nm were taken at designated intervals employing a microplate reader.

### EdU assay

Cells in the logarithmic growth phase were harvested and seeded in 96-well plates at a density of 2 × 10^4 cells per well, followed by incubation in a cell culture incubator for 8–12 hours. Prior to the assay, the medium was exchanged for a fresh solution containing 50 μM 5-Ethynyl-2’-deoxyuridine (EdU; UElandy, China), followed by a 2-hour incubation. The proliferation rate was ultimately defined as the ratio of EdU-positive cells to the total number of nuclei, following image acquisition with a fluorescence microscope.

### Colony formation assay

The clonogenic capacity and long-term proliferation potential of cells were assessed by seeding them in 6-well plates at a density of 1000 cells per well, followed by a 14-day culture period during which the medium was refreshed every third day. After the incubation, colonies were fixed with 4% paraformaldehyde (20 min) and staining with 0.1% crystal violet. Colony counting was performed using ImageJ (v1.8).

### CHX chase assay

For the protein stability assay, cells stably transduced with lentivirus were treated with 25 μg/ml cycloheximide (CHX; HY12320, MedChemExpress) for specified time periods. After treatment, cells were harvested and lysed using cell lysis buffer (87787, Thermo Fisher) supplemented with both protease and phosphatase inhibitors (78442, Thermo Fisher). The resulting lysates were then subjected to Western blot analysis.

### Oxygen consumption rate (OCR) and extracellular acidification rate (ECAR)

Mitochondrial respiration and glycolytic function were measured with an XF96 Extracellular Flux Analyzer. The XF Cell Mito Stress Test and XF Glycolysis Stress Test kits (Seahorse Bioscience, USA) were used in accordance with the manufacturer’s guidelines. Data visualization was carried out using Prism.

### Co-immunoprecipitation and ubiquitination assays

The co-immunoprecipitation (Co-IP) experiment was performed as follows: Cells were lysed with pre-cooled RIPA buffer containing protease and phosphatase inhibitors, followed by supernatant collection (4 °C, 12,000 rpm, 10 min). The supernatant was incubated with 6 μg of primary antibody at 4 °C with gentle shaking overnight. The next day, 40 μL of Protein A/G PLUS-agarose beads (Beyotime, China) were added and incubated at 4 °C with shaking for 8–12 h. After incubation, beads were collected by centrifugation at 2500 rpm for 10 min at 4 °C, and the precipitate was subjected to Western blot analysis. For the ubiquitination assay, cells with FBXO39 overexpression or knockdown were pretreated with the proteasome inhibitor MG132 for 6 hours before lysis. Subsequently, co-immunoprecipitation was performed using an anti-p53 antibody, followed by Western blot analysis with an anti-ubiquitin antibody.

### Subcutaneous xenograft tumor model

Five- to six- week-old male BALB/c nude mice were purchased from Ziyuan Laboratory Animal Technology Co., Ltd. (Hangzhou, China) and randomly divided into experimental groups (*n* = 5 per group). After one week of acclimatization under specific pathogen-free (SPF) conditions, each mouse received a subcutaneous injection in the axilla with 1 × 10^6 CRC cells transduced with FBXO39 knockdown or negative control (NC) lentivirus. Tumor formation was monitored regularly after inoculation, and tumor volume was measured every 5 days. For in vivo imaging, mice were anesthetized with isoflurane and analyzed using an in vivo imaging system (RWD, China). After 6 weeks, all mice were humanely euthanized under isoflurane anesthesia, and tumors were excised for further analysis.

### Chromatin immunoprecipitation quantitative PCR (ChIpqpcr)

ChIPqPCR was performed using p53wildtype CRC cell lines. Cells were crosslinked with 1% formaldehyde for 10 min at room temperature, and the reaction was quenched with glycine. After lysis, chromatin was sheared by sonication to fragments of 200–500 bp. A portion of the sheared chromatin was saved as the “Input” control. The remaining chromatin was incubated overnight at 4 °C with Protein A/G magnetic beads together with either a p53specific antibody (experimental group) or normal rabbit IgG (negative control group). The immunoprecipitated complexes were washed, eluted, and reversecrosslinked at 65 °C overnight. DNA was then purified.

Enrichment of the specific *LDHA* promoter region was detected by qPCR using the following primers:

**Forward:** 5′GTAGAGATGGGGACCCACTG3′

**Reverse:** 5′AGTACTTCGGAAGGCTAAGG3′

The relative enrichment was calculated by the ΔΔCt method, normalizing to the Input control and comparing with the IgG control group0.2.16. **Dual-luciferase Reporter Assay**

To validate the direct regulatory effect of p53 on the activity of the LDHA gene promoter, a dual-luciferase reporter assay was performed. Reporter plasmids containing either the wild-type (WT) or mutant (MUT) LDHA promoter sequence were co-transfected with either a p53 overexpression plasmid or an empty vector control into p53-wild-type CRC cells (HCT116 and RKO). The Renilla luciferase reporter plasmid pRL-TK was co-transfected as an internal control to normalize for transfection efficiency. Forty-eight hours after transfection, both Firefly and Renilla luciferase activities in cell lysates were measured using a dual-luciferase reporter assay kit. The relative transcriptional activity of the LDHA promoter was calculated by normalizing the Firefly luciferase activity to the corresponding Renilla luciferase activity.

### Statistical analysis

Statistical analyses were performed using R (v4.2.2) and GraphPad Prism (v10.1) software. Data are presented as the mean ± standard deviation. Specifically, for comparisons between two groups, two-tailed Student’s t-tests (paired or unpaired, as appropriate) were used for normally distributed data, while the Mann-Whitney U test was applied for data that deviated from normality. Differences in survival curves were assessed with the Log-rank test. *p* < 0.05 was considered statistically significant.

## Results

### Identification of FBXO39 in colorectal cancer and its clinical value as a risk factor

**Fig. 1:**
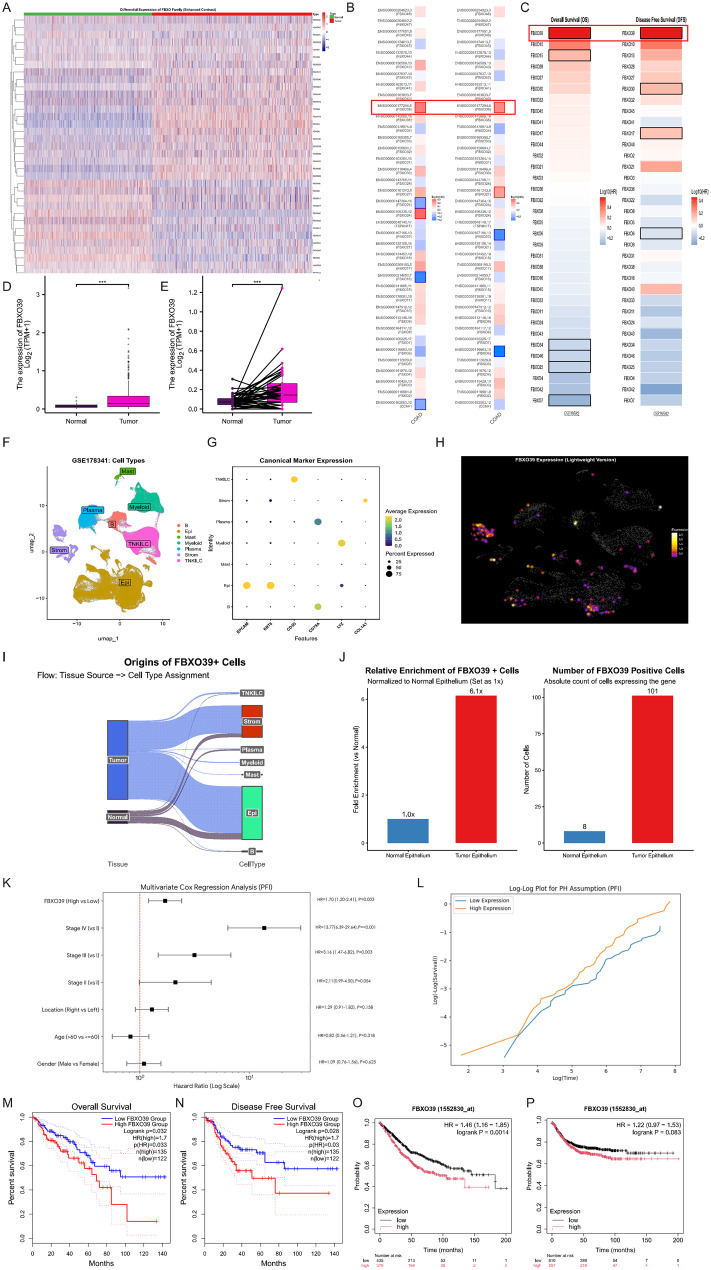
Identification of FBXO39 in colorectal cancer and its clinical value as a risk factor. (**A**) Heatmap of differential expression of FBXO family genes in CRC and normal tissues based on TCGA database. (**B**, **C**) Forest plots visualizing the results of cox regression analysis for overall survival (OS) and disease-free survival (DFS) of the FBXO family based on TCGA and GSE39582 datasets, respectively. (**D**) Comparison of FBXO39 expression levels in CRC tissues versus normal tissues from TCGA data. (**E**) Paired analysis of FBXO39 expression in paired tumor and adjacent normal tissues. (**F**, **G**) Single-cell RNA sequencing analysis showing the t-SNE plot of major cell clusters and their characteristic gene expression patterns in CRC tissues. (**H**) Density plot indicating FBXO39 expression is enriched in stromal and epithelial cells. (**I**) Sankey diagram illustrating that FBXO39 expression primarily originates from intestinal epithelial cells, with the majority derived from tumor cells. (**J**) Quantitative comparison of FBXO39 expression in tumor versus normal intestinal epithelial cells. (**K**) Multivariable cox regression analysis stratified by tumor stage. (**L**) Proportional hazards assumption test for the cox model covariates. (**M**, **N**) Kaplan-meier curves showing the association between high FBXO39 expression and poorer OS and DFS in the TCGA cohort. (**O**, **P**) External validation of the prognostic value of FBXO39 for OS and DFS using the KM-Plotter database. Statistical significance is indicated as * *p* < 0.05, ** *p* < 0.01, *** *p* < 0.001

The F-box only (FBXO) family constitutes a key component of the SCF E3 ubiquitin ligase complex, which regulates diverse biological processes including cell cycle, metabolism, and tumor progression via ubiquitin-mediated protein degradation [14]. To identify FBXO members with potential clinical relevance in CRC, we performed a systematic screening of all 45 FBXO family genes. Based on the TCGA database, we performed differential expression analysis of the 45 FBXO family genes in CRC and visualized a subset of up- and down-regulated members via a heatmap, thereby preliminarily screening FBXO proteins potentially critical in CRC progression (Fig. 1A). Subsequently, to evaluate the prognostic significance of these differentially expressed genes, we used overall survival (OS) and disease-free survival (DFS) as clinical endpoints and performed Cox regression analysis based on TCGA data (Fig. 1B) and the GEO dataset GSE39582 (Fig. 1C), respectively, to identify risk and protective factors associated with prognosis. The results showed that FBXO39 was the only FBXO gene identified as an independent risk factor in both analyses. Based on the above findings, we further investigated the expression of FBXO39 in CRC. Analysis of TCGA data showed that the overall expression level of FBXO39 was significantly elevated in CRC tissues compared to normal tissues (Fig. 1D). Paired comparative analysis between tumor and normal tissues further confirmed that FBXO39 expression was significantly upregulated in tumors (Fig. 1E). To dissect the expression distribution of FBXO39 at the single-cell level, we analyzed single-cell RNA sequencing data. The cell cluster map displays the distribution of major cell types in CRC tissues (Fig. 1F) and their characteristic gene expression (Fig. 1G). A density plot suggests that FBXO39 is mainly expressed in stromal cells and epithelial cells (Fig. 1H). A Sankey diagram further reveals that FBXO39 expression primarily originates from intestinal epithelial cells, with the vast majority derived from tumor cells (Fig. 1I). Quantitative comparison showed that within intestinal epithelial cells, FBXO39 expression in tumor cells was 6.1 times higher than in normal epithelial cells (Fig. 1J). To systematically evaluate the prognostic value of FBXO39, we performed univariate and multivariate Cox regression analyses based on TCGA data. The results indicated that FBXO39 could serve as an independent prognostic risk factor (Table 2). Further stratified analysis by tumor stage showed that high FBXO39 expression retained significant prognostic value in stage III and IV patients, while no significant association was observed in stage I and II patients (Fig. 1K). The proportional hazards assumption test indicated that the covariates met the proportional hazards assumption (Fig. 1L). Finally, we validated the prognostic significance of FBXO39 through survival analysis. Analysis based on TCGA data demonstrated that high FBXO39 expression was significantly associated with poorer OS and DFS (Fig. 1M, N). To independently verify this conclusion, we used the KM-plotter database as an external validation set, and the results similarly showed that high FBXO39 expression was significantly associated with worse OS and DFS (Fig. 1O, P). In summary, we identified FBXO39 as an independent risk factor with abnormally elevated expression in CRC, and its expression level consistently correlates with poor patient prognosis.

**Table 2:** Univariate and multivariate cox analysis for OS (TCGA cohort)

Characteristics	Univariate Analysis HR (95% CI)	P-value	Multivariate Analysis HR (95% CI)	P-value
**Age** (>60 vs ≤ 60)	0.70 (0.49–0.99)	0.042	0.82 (0.56–1.21)	0.318
**Gender** (Male vs Female)	1.25 (0.90–1.73)	0.185	1.09 (0.76–1.56)	0.625
**Tumor Location** (Right vs Left)	0.95 (0.68–1.33)	0.758	1.29 (0.91–1.82)	0.158
**T Stage** (T3-4 vs T1-2)	3.11 (1.68–5.75)	<0.001	–	–
**N Stage** (Positive vs Negative)	2.72 (1.96–3.78)	<0.001	–	–
**M Stage** (M1 vs M0)	6.88 (4.77–9.93)	<0.001	–	–
**Stage** (AJCC)				
Stage I	Reference		Reference	
Stage II	2.02 (0.95–4.28)	0.068	2.11 (0.99–4.50)	0.054
Stage III	3.13 (1.46–6.71)	0.003	3.16 (1.47–6.82)	0.003
Stage IV	14.56 (6.88–30.80)	<0.001	13.77 (6.39–29.64)	<0.001
**FBXO39** (High vs Low)	1.95 (1.40–2.71)	<0.001	1.70 (1.20–2.41)	0.003

### FBXO39 expression is upregulated in CRC

**Fig. 2:**
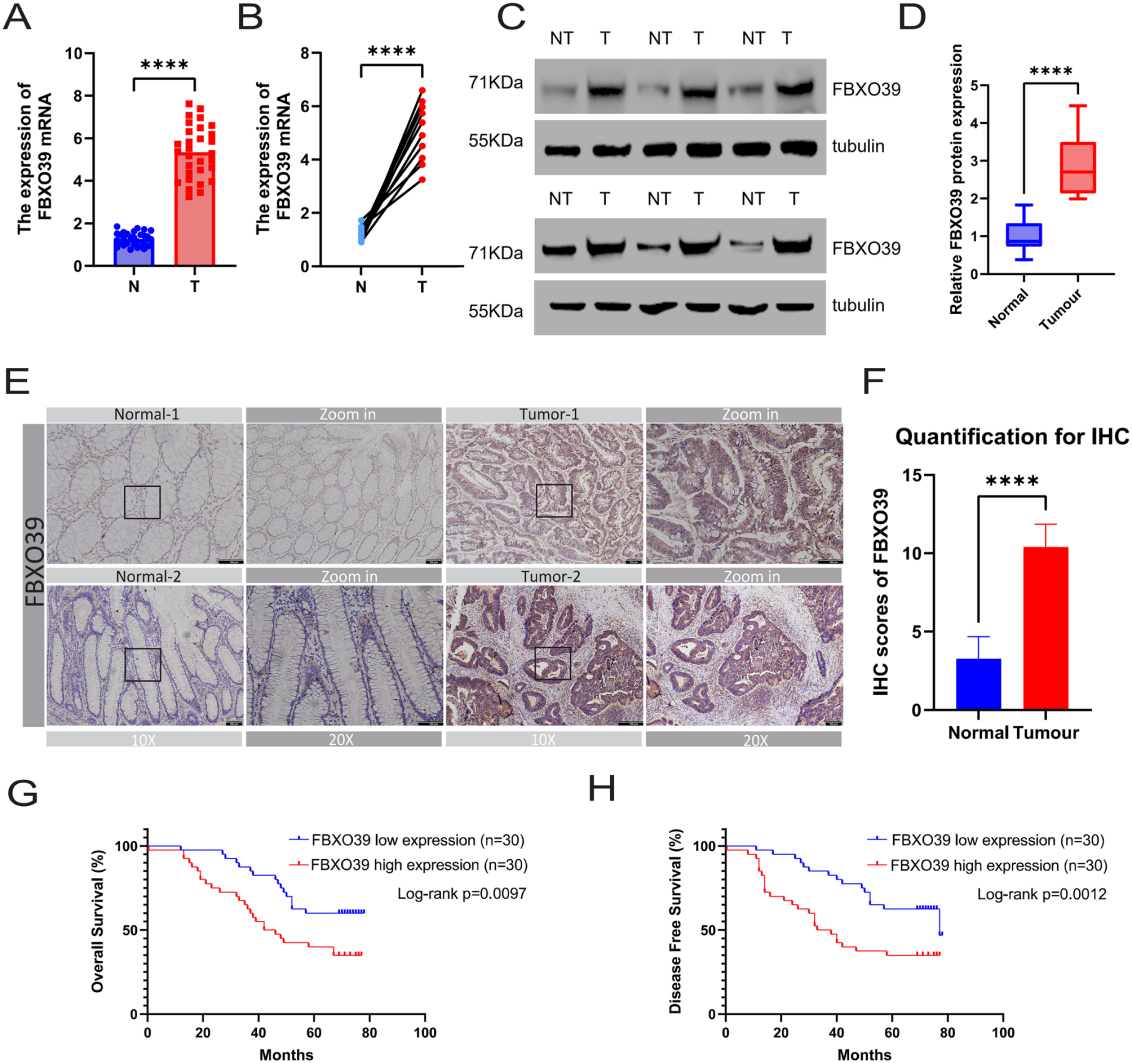
FBXO39 expression is upregulated in CRC. (**A**,**B**) qRT-PCR analysis of FBXO39 mRNA expression in unpaired and paired CRC tissues versus adjacent normal tissues from 30 clinical samples. (**C**,**D**) Western blot analysis and quantification of FBXO39 protein levels in paired clinical samples. (**E**,**F**) Immunohistochemistry (IHC) analysis of FBXO39 expression in clinical specimens, including representative images and quantitative IHC scores. (**G**,**H**) analysis of OS and DFS in patients with high versus low FBXO39 expression in the retrospective cohort from the Second affiliated hospital of Nanchang University. Data are presented as mean ± standard deviation. ***p < 0.001

We detected FBXO39 expression levels in 30 pairs of collected CRC and matched normal tissue samples. qRT-PCR analysis revealed that the mRNA expression level of FBXO39 in CRC tissues was significantly higher than in paired normal tissues (Fig. 2A, B). Furthermore, Western blotting analysis indicated that the protein expression level of FBXO39 was also markedly upregulated in CRC tissues compared to adjacent normal tissues (Fig. 2C, D). Subsequently, immunohistochemistry also confirmed the upregulation of FBXO39 in CRC tissues (Fig. 2E, F). Finally, we evaluated data from a retrospectively collected CRC cohort at the Second Affiliated Hospital of Nanchang University and found that patients with high FBXO39 expression had poorer OS and DFS than patients with low FBXO39 expression (Fig. 2G, H). In conclusion, FBXO39 is upregulated in CRC and associated with a worse prognosis.

### FBXO39 enhances CRC cell growth both in vitro and in vivo

**Fig. 3:**
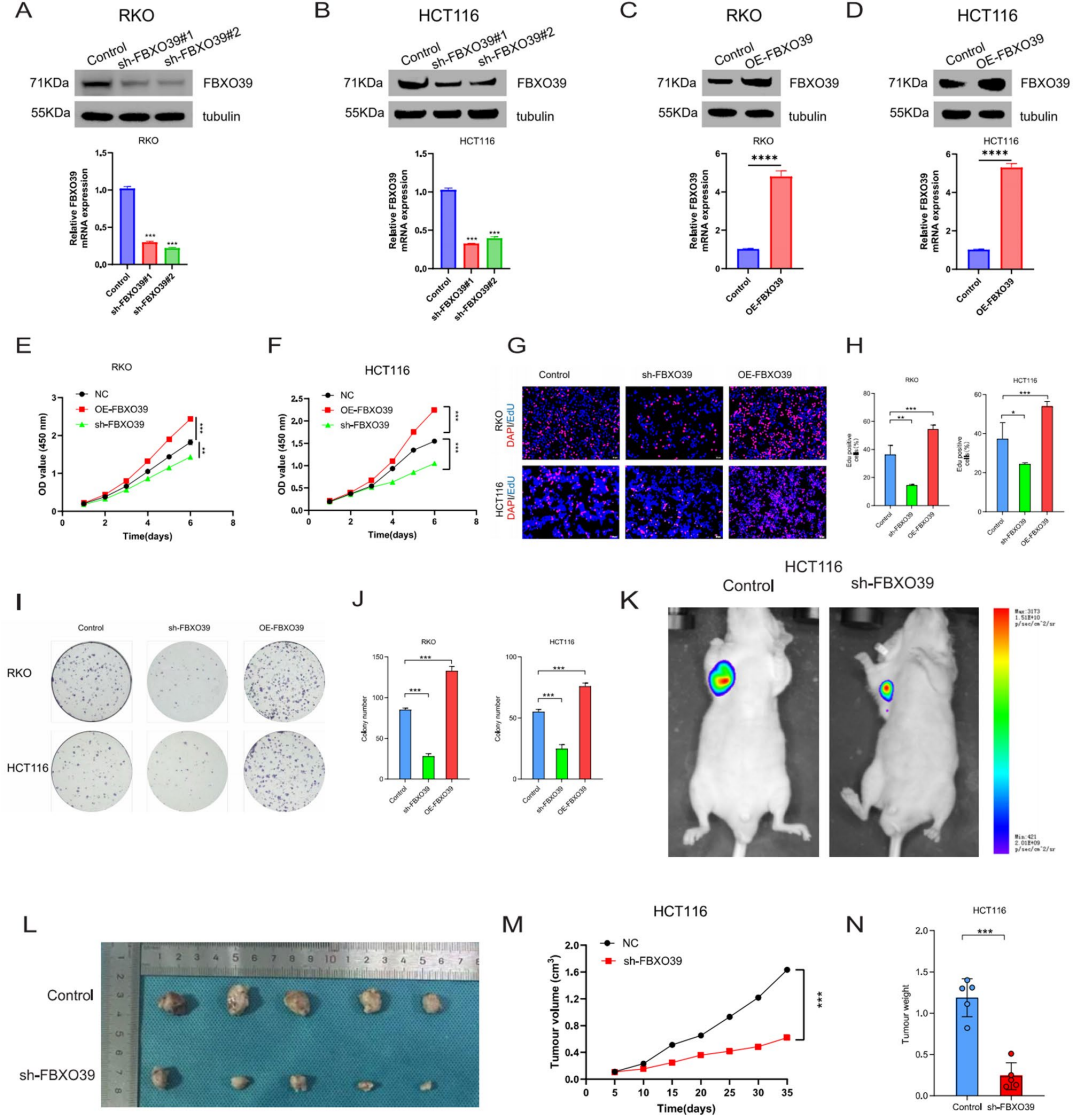
FBXO39 promotes CRC cell proliferation in vitro and in vivo. (**A**-**D**) efficiency of FBXO39 knockdown and overexpression assessed by qRT-PCR and Western blot in p53-wild type CRC cell lines RKO and HCT116. (**E**,**F**) proliferation curves of RKO and HCT116 cells measured by CCK-8 assay. (**G**,**H**) cell proliferation activity evaluated by EdU incorporation assay. (**I**,**J**) long-term proliferation capacity assessed by colony formation assay. (**K**-**N**) in vivo tumor growth analyzed via tumor growth curves, representative images, and tumor weight in a subcutaneous xenograft model. Data are presented as mean ± standard deviation (***p < 0.001)

We constructed RKO and HCT116 cell lines with stable FBXO39 knockdown and overexpression for subsequent experiments to investigate the specific function of FBXO39 in CRC. These two cell lines are also p53-wild type cells in CRC (Fig. 3A–D). CCK-8 assay results showed that FBXO39 overexpression promoted CRC cell growth, while FBXO39 knockdown inhibited their growth (Fig. 3E, F). EdU assay results further demonstrated that FBXO39 overexpression enhanced the proliferative capacity of CRC cells, while FBXO39 knockdown exhibited proliferation inhibitory effects (Fig. 3G, H). Additionally, colony formation assays revealed that the OE-FBXO39 group formed more cell colonies than the control group, while the sh-FBXO39 group showed significantly reduced colony numbers (Fig. 3I, J). Finally, through subcutaneous xenograft model validation, we found that compared with the control group, tumor growth rate was significantly slowed in sh-FBXO39 group mice, with both final average tumor volume and average weight markedly reduced (Fig. 3K–N). In summary, FBXO39 demonstrated effects in promoting CRC progression in both in vitro and in vivo experiments.

### FBXO39 reduces p53 protein levels and directly interacts with p53 in CRC cells with wild-type p53

**Fig. 4:**
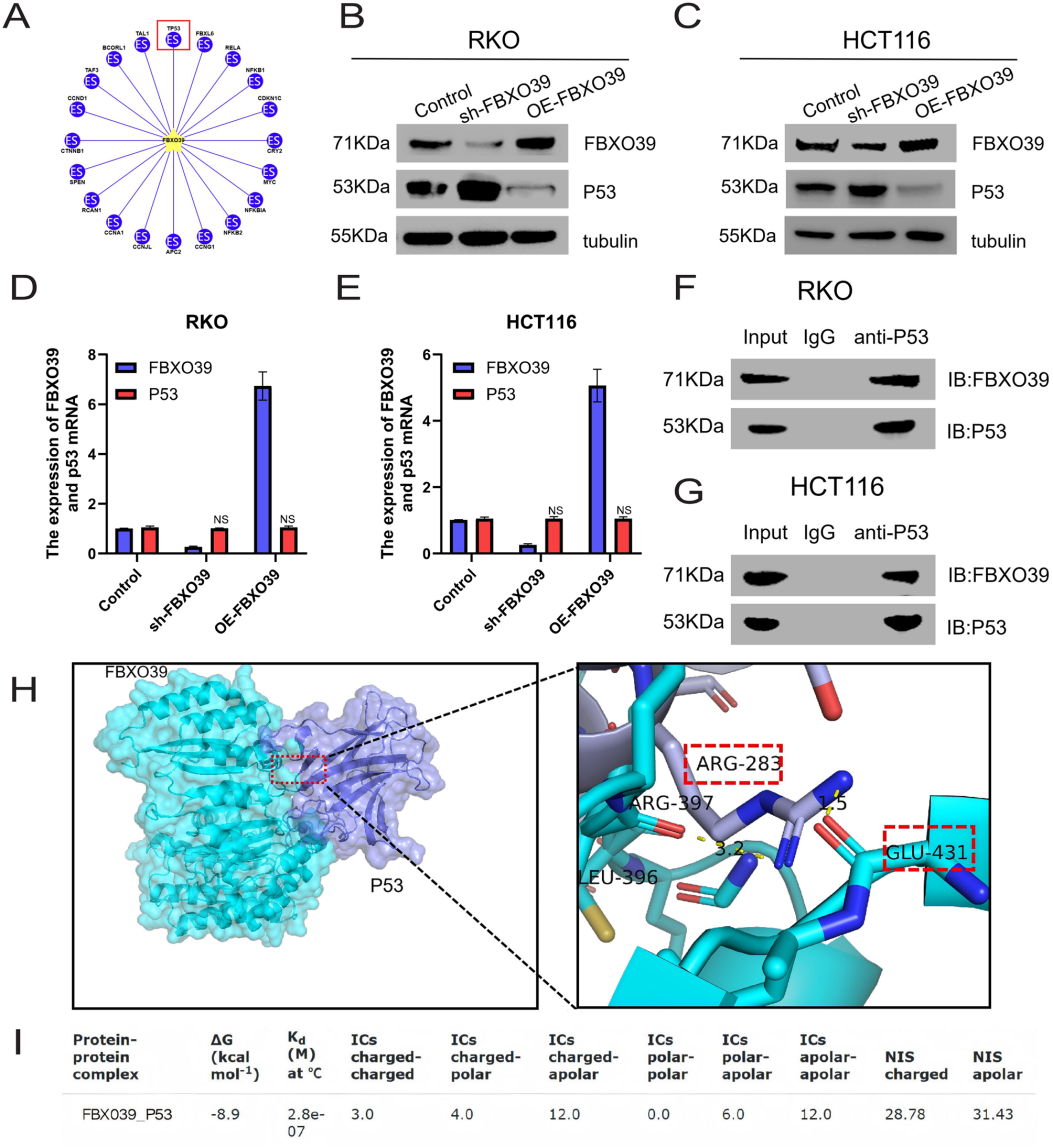
FBXO39 decreases p53 protein levels and directly interacts with p53. (**A**) prediction of the interaction between FBXO39 and p53 using ubibrowser 2.0 software. (**B**, **C**) Western blot analysis of p53 protein levels after knockdown or overexpression of FBXO39 in RKO and HCT116 cells. (**D**, **E**) detection of TP53 mRNA levels after intervention of FBXO39 in RKO and HCT116 cells. (**F**, **G**) Co-immunoprecipitation (co-IP) assay validating the binding between FBXO39 and p53 in RKO and HCT116 cells. (**H**) predicted binding interface between FBXO39 and p53 from molecular docking. (**I**) corresponding binding energy for the FBXO39-p53 interaction model

We employed Ubibrowser 2.0 software for predictive analysis of potential FBXO39 binding proteins, with results indicating that FBXO39 may interact with the tumor suppressor p53 (Fig. 4A). We subsequently further verified whether FBXO39 regulates p53 expression. Western blot experiments demonstrated that upregulating FBXO39 expression decreased p53 protein levels, whereas downregulating FBXO39 increased p53 protein expression (Fig. 4B, C). However, changes in FBXO39 expression did not significantly affect p53 mRNA levels (Fig. 4D, E). To further investigate whether FBXO39 directly interacts with p53 in CRC cells, we performed co-immunoprecipitation (co-IP) assays, which confirmed a binding relationship between the two proteins (Fig. 4F, G). Furthermore, molecular docking analysis supported their direct interaction, with Fig. 4H depicting the binding sites from protein–protein docking and Fig. 4I showing the corresponding binding energy. In summary, this study discovered that in p53-wild type RKO and HCT116 CRC cells, FBXO39 can reduce p53 protein levels and directly bind to p53.

### FBXO39 facilitates the degradation of p53 protein through the ubiquitin-proteasome pathway

**Fig. 5:**
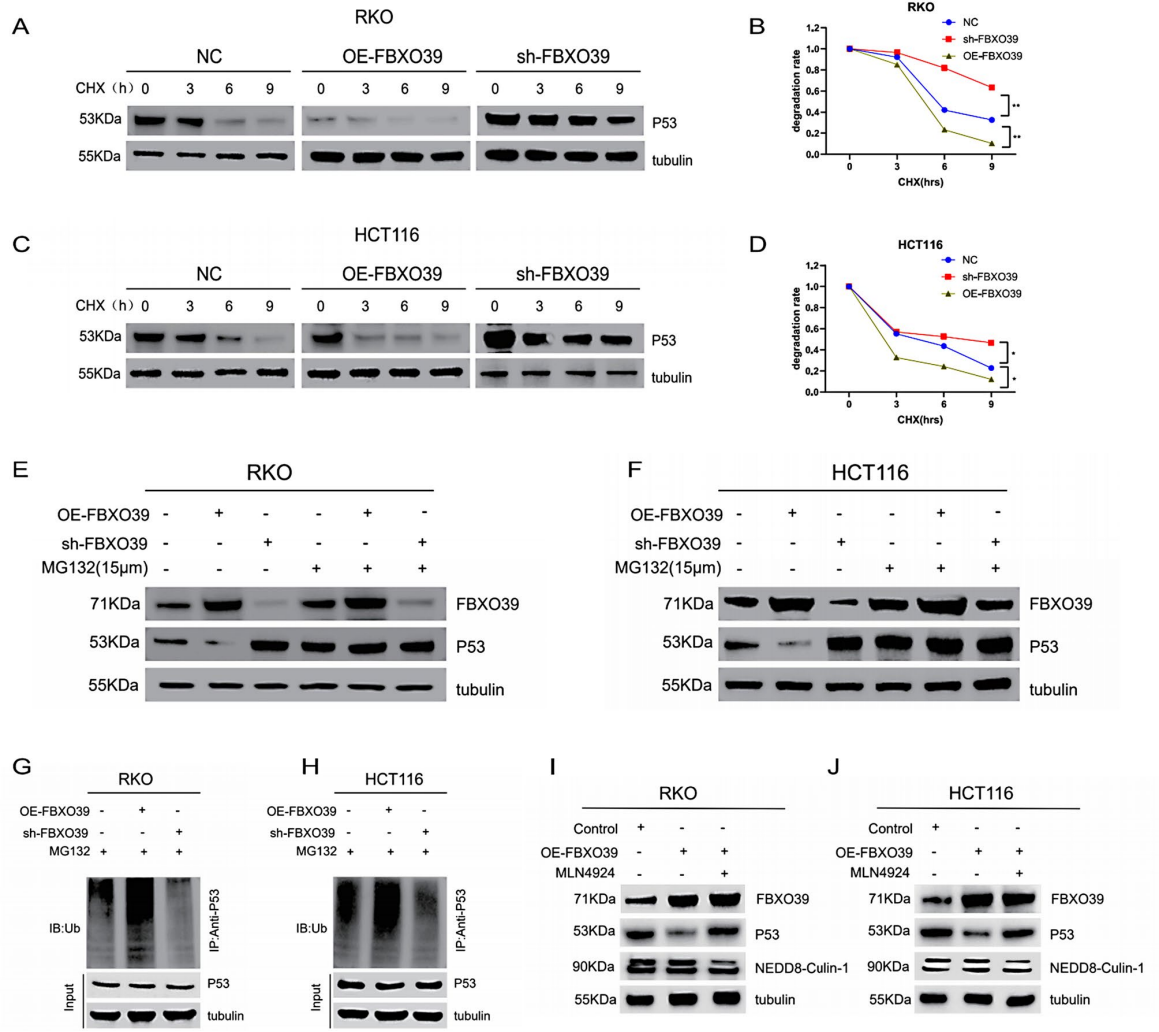
FBXO39 promotes p53 protein degradation via the ubiquitin-proteasome pathway. (**A**-**D**) degradation kinetics of p53 protein detected by Western blot after treatment with the protein synthesis inhibitor cycloheximide (CHX, 25 µg/mL) at different time points in RKO and HCT116 cells. (**E**, **F**) changes in p53 protein levels before and after FBXO39 manipulation assessed by treating cells with the proteasome inhibitor MG132 (15 µM). (**G**, **H**) effect of FBXO39 on p53 ubiquitination analyzed by co-immunoprecipitation to detect p53 polyubiquitination levels in the presence of MG132 (15 µM). (**I**, **J**) Western blot analysis of p53 protein levels and NEDD8CUL1 conjugation in RKO and HCT116 cells overexpressing FBXO39, following treatment with or without the E3 ubiquitin ligase inhibitor MLN4924 (0.5 µM, 24 h). Data are presented as mean ± standard deviation (*p < 0.05, **p < 0.01)

We conducted further investigations to elucidate the specific molecular mechanism by which FBXO39 regulates p53 expression in p53-WT CRC cells. Our existing results indicated that FBXO39 directly binds p53 and downregulates its protein expression without significantly affecting its mRNA level, suggesting FBXO39 might regulate p53 protein stability through post-translational modification pathways. Given that FBXO39 belongs to the E3 ubiquitin ligase family, we hypothesized it might mediate ubiquitin-dependent degradation of p53. First, we assessed p53 protein stability using a cycloheximide chase assay. Results showed that FBXO39 overexpression significantly promoted p53 protein degradation, whereas FBXO39 knockdown effectively delayed the p53 degradation process (Fig. 5A–D). To further verify whether FBXO39 regulates p53 through the proteasomal pathway, we treated cells with the proteasome inhibitor MG132. Results demonstrated that in control cells, MG132 caused p53 protein accumulation, indicating basal levels of proteasomal degradation of p53; in FBXO39-overexpressing cells, MG132 completely blocked the FBXO39-induced p53 decrease; whereas in FBXO39-knockdown cells, where p53 was already at elevated levels, MG132 treatment did not cause further accumulation (Fig. 5E,F). These results indicate that FBXO39 regulates p53 stability through the proteasomal pathway. Furthermore, we detected p53 ubiquitination levels via an in vivo ubiquitination assay. Using a p53 antibody for co-immunoprecipitation, we found that FBXO39 overexpression significantly enhanced p53 polyubiquitination, whereas FBXO39 knockdown markedly attenuated this modification (Fig. 5G,H). To provide further evidence that FBXO39 directly regulates p53 ubiquitination through its E3 ligase activity, we employed MLN4924 for intervention. MLN4924 is a selective NEDD8-activating enzyme inhibitor that blocks the activity of CRL-type E3 ubiquitin ligases by inhibiting the neddylation of Cullin proteins. In RKO cells, FBXO39 overexpression led to decreased p53 protein levels, and co-treatment with MLN4924 reversed this effect (Fig. 5I). Concurrently, we detected that MLN4924 treatment effectively inhibited the binding of NEDD8 to CULIN-1, confirming the successful pharmacological action of the inhibitor in the experimental system (Fig. 5I). Repetition of the experiment in the HCT116 cell line yielded entirely consistent results (Fig. 5J). In summary, FBXO39, as an E3 ubiquitin ligase, reduces p53 protein levels in p53-WT CRC cells by promoting p53 polyubiquitination and subsequent proteasomal degradation.

### FBXO39 enhances aerobic glycolysis in CRC cells

**Fig. 6:**
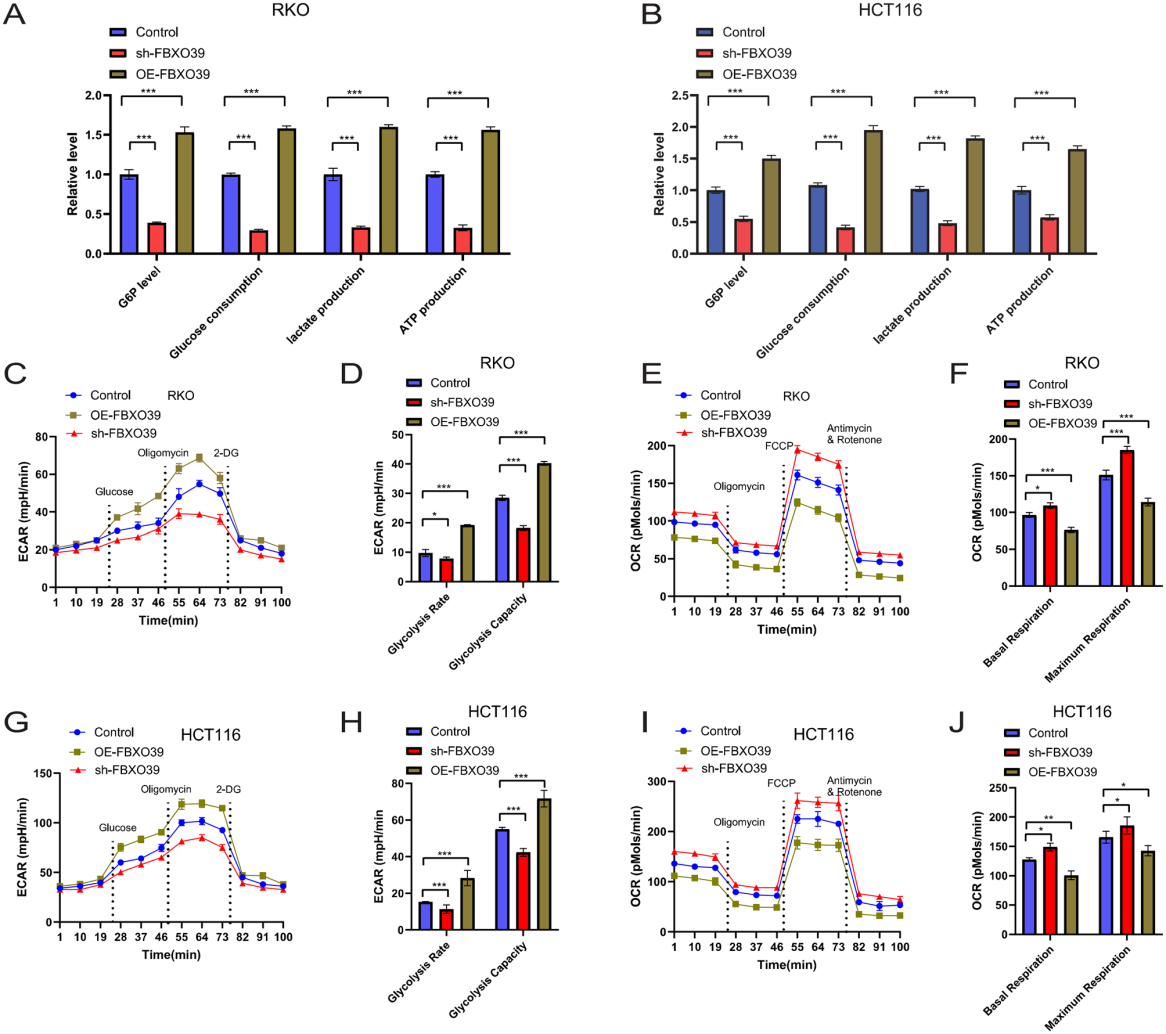
FBXO39 enhances aerobic glycolysis in CRC cells. (**A**, **B**) analysis of glucose metabolism parameters including glucose consumption, G6P levels, lactate production, and ATP concentration in HCT116 and RKO cells. (**C**, **D**, **G**, **H**) extracellular acidification rate (ECAR) to measure glycolytic flux and glycolytic capacity, with sequential addition of glucose (10 mM), oligomycin (1.0 µM), and 2-deoxyglucose (2-DG, 50 mM) at specified time points. (**E**, **F**, **I**, **J**) oxygen consumption rate (OCR) to assess mitochondrial respiratory function, with sequential addition of oligomycin (1.0 µM), the mitochondrial uncoupler FCCP (1.0 µM), and a mixture of rotenone/antimycin a inhibitors (Rote/AA, 0.5 µM) at specified time points. Data are presented as mean ± standard deviation, with statistical significance indicated as *p < 0.05, **p < 0.01, ***p < 0.001

Multiple studies have reported that wild-type p53 can inhibit glycolysis and promote oxidative phosphorylation, thereby exerting tumor-suppressive effects by inhibiting the Warburg effect [26–28]. We therefore hypothesized that FBXO39 might promote CRC progression by regulating aerobic glycolysis. We tested this hypothesis by measuring key parameters of glucose metabolism. Results showed that FBXO39 overexpression promoted glucose uptake, glucose-6-phosphate (G6P) accumulation, lactate secretion, and ATP production in RKO and HCT116 cells; whereas FBXO39 knockdown reduced the levels of these metabolites (Fig. 6A, B), indicating that FBXO39 promotes glucose metabolism. We assessed the impact of FBXO39 on total glycolytic flux by sequentially adding glucose, oligomycin, and 2-deoxyglucose (2-DG) at different time points and measuring the extracellular acidification rate (ECAR) to reflect glycolytic flux. Results demonstrated that FBXO39 overexpression significantly increased ECAR, while its knockdown decreased ECAR (Fig. 6C, D, G, H). Additionally, we evaluated mitochondrial respiration by measuring the oxygen consumption rate (OCR), sequentially adding the oxidative phosphorylation inhibitor oligomycin, the mitochondrial uncoupler carbonyl cyanide-p-trifluoromethoxyphenylhydrazone (FCCP), and a mixture of the mitochondrial complex I inhibitor rotenone and the mitochondrial complex III inhibitor antimycin A (Rote/AA). We found that FBXO39 overexpression inhibited both basal and maximal mitochondrial respiration, whereas its knockdown promoted these two types of respiration (Fig. 6E, F, I, J). In summary, FBXO39 promotes aerobic glycolysis in CRC cells while simultaneously inhibiting mitochondrial respiration.

### FBXO39 promote tumor cell growth and aerobic glycolysis by increasing LDHA expression via p53 in p53-wild type CRC cells

**Fig. 7:**
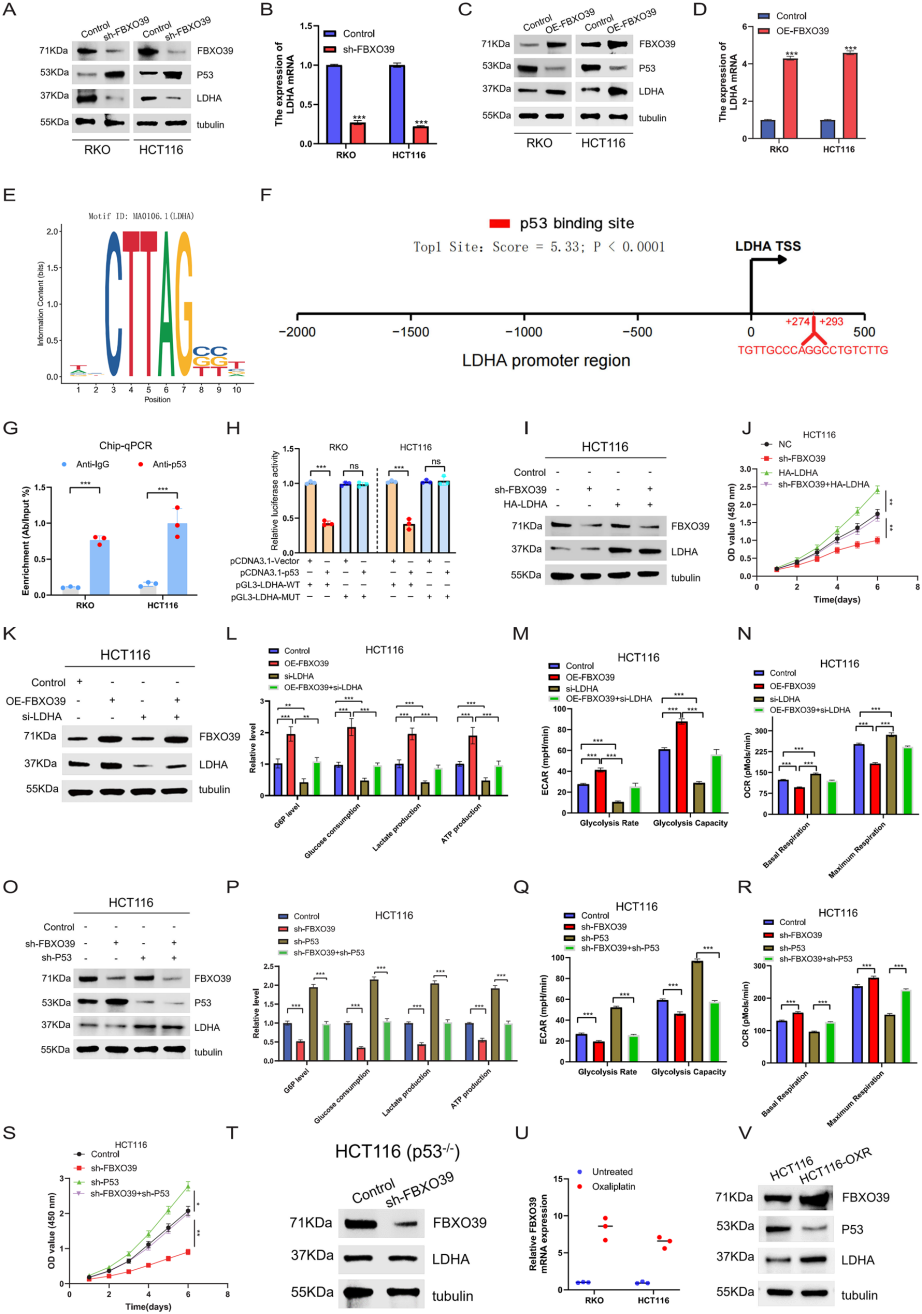
FBXO39 promote tumor cell growth and aerobic glycolysis by increasing LDHA expression via p53 in p53-wild type CRC cells. (**A**, **B**) expression of LDHA at mRNA and protein levels after knocking down FBXO39 in p53-wild type HCT116 and RKO cells. (**C**, **D**) expression of LDHA at mRNA and protein levels after overexpressing FBXO39 in HCT116 and RKO cells. (**E**) schematic of potential p53 binding motifs in the LDHA promoter region predicted by JASPAR database analysis. (**F**) diagram of the most probable p53 binding site located upstream of the LDHA transcription start site. (**G**) chromatin immunoprecipitation-quantitative PCR (ChIP-qPCR) analysis of p53 binding to the LDHA promoter. (**H**) luciferase reporter assay measuring the activity of wild-type (WT) or mutant (MUT) LDHA promoter constructs upon p53 overexpression. (**I**) Western blot confirming HA-LDHA overexpression in the context of FBXO39 knockdown for rescue experiments. (**J**) cell proliferation assessed by CCK-8 assay in HCT116 cells with FBXO39 knockdown, with or without HA-LDHA overexpression rescue. (**K**) Western blot confirming LDHA knockdown efficiency in the context of FBXO39 overexpression. (**L**) measurements of key glycolytic parameters (glucose uptake, G6P accumulation, lactate secretion, ATP production) normalized to total protein content after 72 hours of treatment in the indicated groups (*n* = 3). (**M**) extracellular acidification rate (ECAR) profiles to assess glycolytic flux and capacity, with sequential injection of glucose (10 mM), oligomycin (1.0 µM), and 2-deoxyglucose (2-DG, 50 mM) at 19, 46, and 73 min, respectively. (**N**) OCR profiles to assess mitochondrial basal and maximal respiration, with sequential injection of oligomycin (1.0 µM), the mitochondrial uncoupler FCCP (1.0 µM), and a mixture of rotenone and antimycin a (Rote/AA, 0.5 µM each) at 19, 46, and 73 min, respectively. (**O**) Western blot analysis of LDHA protein levels after concurrent knockdown of FBXO39 and p53. (**P**) measurements of glycolytic parameters (as in L) in cells with FBXO39 knockdown, with or without concurrent p53 knockdown (*n* = 3). (**Q**) ECAR profiles and (**R**) OCR profiles under the conditions described in (**P**), measured with the same sequential injection protocols and time points as in (**M**) and (**N**), respectively. (**S**) cell proliferation assessed by CCK-8 assay in cells with FBXO39 knockdown, with or without concurrent p53 knockdown. (**T**) LDHA protein levels after FBXO39 knockdown in p53-deficient HCT116 (p53-/-) cells. (**U**) FBXO39 mRNA levels in RKO and HCT116 cells after treatment with oxaliplatin (15 µM and 10 µM, respectively) for 72 hours (*n* = 3). (**V**) protein levels of FBXO39, p53, and LDHA in parental and oxaliplatin-resistant (HCT116-OXR) cells. Data are presented as mean ± standard deviation. Statistical significance was determined by Student’s t-test or one-way ANOVA (*p < 0.05, **p < 0.01, ***p < 0.001)

Lactate dehydrogenase A (LDHA) is a key rate-limiting enzyme in aerobic glycolysis. It catalyzes the conversion of pyruvate to lactate while regenerating NAD^+^, thereby ensuring the high-speed and continuous operation of glycolysis to support the malignant proliferation needs of tumor cells [29, 30]. Notably, LDHA has been confirmed as an important downstream target of wild-type p53 in regulating glycolysis [31]. Based on this, we hypothesized that in p53-wild type CRC cells, FBXO39 might downregulate LDHA expression levels through the p53 signaling pathway, thereby inhibiting tumor cell aerobic glycolysis and growth. We first investigated the regulatory role of FBXO39 on LDHA expression. Knocking down FBXO39 in p53-wild type CRC cells HCT116 and RKO significantly downregulated LDHA at both mRNA and protein levels (Fig. 7A, B). Conversely, overexpressing FBXO39 significantly upregulated LDHA expression (Fig. 7C, D). To dissect the transcriptional regulatory mechanism of LDHA, we analyzed its promoter region using the JASPAR database and identified potential p53 binding motifs (Fig. 7E). Among these, the site located at positions +274 to + 293 upstream of the transcription start site was the most probable binding site (Fig. 7F). To verify whether p53 directly binds to this site, we performed chromatin immunoprecipitation-quantitative PCR (ChIP-qPCR). The results showed that, compared to the IgG control, the LDHA promoter fragment was significantly enriched by the p53 antibody (Fig. 7G), demonstrating specific binding of p53 to this region. A further luciferase reporter assay confirmed the specificity of this interaction: when p53 was overexpressed, the activity of the LDHA promoter containing the wild-type (WT) p53 binding site was significantly inhibited, and this inhibitory effect was abolished when the binding site was mutated (MUT) (Fig. 7H). These results directly demonstrate that p53 inhibits LDHA transcription by binding to a specific region of its promoter. Subsequently, we explored the functional impact of this regulatory pathway. Knocking down FBXO39 in HCT116 cells significantly inhibited cell proliferation, and overexpressing LDHA rescued this phenotype (Fig. 7I, J). Next, to more directly demonstrate that FBXO39 mediates its function through LDHA, we performed a rescue experiment. Western blot confirmed successful LDHA knockdown in the context of FBXO39 overexpression (Fig. 7K). Metabolic analysis showed that FBXO39 overexpression significantly promoted the production of key glycolytic metabolites (Fig. 7L) and both glycolytic rate and capacity (ECAR) (Fig. 7M), while inhibiting mitochondrial basal and maximal respiratory oxygen consumption rates (OCR) (Fig. 7N). Concurrent knockdown of LDHA completely blocked these metabolic changes induced by FBXO39 overexpression (Fig. 7L, M, N). Next, to clarify the central role of p53 in this regulatory pathway, we interfered with p53 expression while knocking down FBXO39. Western blot results showed that p53 deletion significantly attenuated the inhibitory effect of FBXO39 knockdown on LDHA expression (Fig. 7O). At the metabolic level, the glycolytic inhibitory phenotypes induced by FBXO39 knockdown, including reduced glucose uptake, G6P accumulation, lactate production, and ATP yield, were all reversed by concurrent p53 knockdown (Fig. 7P). Furthermore, the decreased ECAR (Fig. 7Q) and increased OCR (Fig. 7R) caused by FBXO39 knockdown were also partially counteracted by p53 knockdown. Additionally, p53 knockdown partially restored the impaired cell proliferation capacity resulting from FBXO39 knockdown (Fig. 7S). Notably, in p53-deficient HCT116 p53-/- cells, knocking down FBXO39 had no significant effect on LDHA expression (Fig. 7T), further confirming the p53-dependence of this regulatory pathway. Finally, we examined the relationship between FBXO39 and chemotherapy drugs to explore its potential clinical significance. After treating cells with oxaliplatin (RKO IC₅₀ ≈ 15 μM, HCT116 IC₅₀ ≈ 10 μM) for 72 hours, FBXO39 mRNA levels increased significantly (Fig. 7U). Moreover, in our self-established oxaliplatin-resistant cell line (HCT116-OXR), we observed elevated FBXO39 expression accompanied by decreased p53 levels and increased LDHA levels (Fig. 7V), suggesting the FBXO39-p53-LDHA axis may play a role in chemotherapy resistance. In summary, in p53 wild-type CRC, FBXO39 downregulates LDHA expression through a p53-dependent transcriptional repression mechanism, thereby inhibiting tumor cell aerobic glycolytic activity and proliferation capacity.

## Discussion

CRC represents a significant global health burden, with its high morbidity and mortality rates continuously challenging healthcare systems worldwide [32]. Patients with advanced disease often experience poor prognosis due to chemotherapy resistance and suboptimal responses to immunotherapy [33, 34], highlighting the importance of elucidating the molecular mechanisms underlying CRC development and progression. This study employed systematic bioinformatics approaches to analyze the expression and prognosis of FBXO family genes in CRC, aiming to identify members with the most significant prognostic value.

We thereby identified FBXO39 as a novel oncogene critical for CRC progression. FBXO39 belongs to the E3 ubiquitin ligase family and plays a key role in regulating protein stability [35, 36]. In recent years, multiple bioinformatics studies have reported abnormal FBXO39 expression in various malignancies. Research indicates that FBXO39 is significantly overexpressed in glioma, breast cancer, cervical squamous cell carcinoma, and osteosarcoma, and is closely associated with poor patient prognosis [37–39]. For instance, high FBXO39 expression was detectable in serum exosomes from 86% of breast cancer patients, with its expression levels correlating with clinical stage and HER2 status [38]. Although some studies have reported FBXO39 upregulation in CRC, these were limited to expression detection without in-depth exploration of its biological functions. Overall, existing research on FBXO39 predominantly remains confined to bioinformatics analyses or clinical correlation studies, generally regarding it as an oncogene but lacking mechanistic insights into its specific pro-tumorigenic actions. Based on this, we systematically investigated the biological role of FBXO39 in CRC. Our results demonstrated significantly elevated FBXO39 expression in CRC tissues, with high expression correlating with poorer OS and DFS in patients. Furthermore, both in vitro and in vivo experiments indicated that FBXO39 substantially enhances the proliferative capacity of CRC cells. These findings suggest that FBXO39 may serve as a novel biomarker for poor prognosis in CRC and exerts oncogenic functions in this malignancy.

Metabolic reprogramming represents a key mechanism enabling tumor cells to adapt to hostile microenvironments and sustain rapid proliferation [40]. Enhanced aerobic glycolysis serves as a critical promoter of CRC progression and chemotherapy resistance. Recent years have witnessed numerous studies dedicated to unraveling the specific regulatory mechanisms of aerobic glycolysis in CRC [41]. For example, METTL3 promotes aerobic glycolysis and tumor cell proliferation by stabilizing mRNAs of glycolysis-related genes HK2 and GLUT1 through m6A modification [42]; PRMT1 enhances glycolytic flux by methylating PGK1 to augment its phosphorylation activity [43]; UBTD1 drives aerobic glycolysis and tumor progression by stabilizing c-Myc and upregulating HK2 expression [44]. In this study, we established the crucial role of FBXO39 in aerobic glycolysis in CRC, demonstrating its ability to promote aerobic glycolysis while simultaneously inhibiting mitochondrial respiration in CRC cells. This finding underscores the significant role of FBXO39 in glucose metabolism, suggesting its potential as a therapeutic target in CRC.

To further investigate the specific mechanisms through which FBXO39 regulates aerobic glycolysis, we focused our research on the p53 protein. As a central regulator of cellular metabolism, p53 profoundly influences tumorigenesis through its functional activities. Regarding aerobic glycolysis, p53 negatively regulates this process through multiple direct and indirect transcriptional regulatory pathways, thereby exerting its tumor-suppressive effects [45, 46]. Recent studies have revealed that p53‘s metabolic regulatory functions are precisely controlled by various post-translational modifications. For instance, pyrimidine synthase CAD mediates p53 deamidation at asparagine residues N235 and N239, directly leading to loss of its transcriptional activity [47]; the E3 ubiquitin ligase TRIM33 promotes K48-linked polyubiquitination and degradation of p53, resulting in loss of its glycolytic inhibitory function and ultimately promoting tumorigenesis [31]; lactate in the tumor microenvironment can inhibit p53‘s DNA-binding capacity and impair its tumor-suppressive function through AARS1-mediated p53 lactylation [48]. However, in CRC harboring wild-type TP53, p53 protein frequently exhibits low expression levels, and the underlying mechanisms remain incompletely understood [49]. We explored this mechanism through a series of experiments: first, Co-IP and molecular docking experiments confirmed the interaction between FBXO39 and p53; subsequently, investigations revealed that FBXO39 promotes p53 protein degradation via ubiquitination; ultimately, we demonstrated that FBXO39 promotes aerobic glycolysis and inhibits mitochondrial respiration in CRC cells by downregulating p53. These results provide compelling evidence that FBXO39 promotes aerobic glycolysis and proliferative capacity in CRC cells by ubiquitinating and degrading wild-type p53.

Lactate dehydrogenase A (LDHA) is the key enzyme catalyzing the reduction of pyruvate to lactate while oxidizing NADH generated during glycolysis. It acts as a rate-limiting enzyme in the final step of glycolysis, ensuring high-speed glycolytic flux and providing energy for malignant tumor proliferation [30, 50]. For example, NCAPD3 promotes glycolysis and proliferation in CRC cells by upregulating LDHA transcription through c-MYC [51]. Sodium butyrate inhibits aerobic glycolysis and cell proliferation in CRC by suppressing HIF-1α-mediated LDHA expression [52]. LDHA also serves as a crucial downstream target of p53 in glycolytic regulation, as p53 can downregulate LDHA expression through transcriptional repression or other mechanisms [53].

From a therapeutic perspective, the findings of this study also suggest potential intervention strategies. Our experiments revealed that oxaliplatin treatment induces upregulation of FBXO39 expression, and significant alterations in the FBXO39-p53-LDHA pathway were observed in oxaliplatin-resistant cells, implying this pathway may be involved in chemotherapeutic stress adaptation and the drug-resistant phenotype. However, directly targeting E3 ubiquitin ligases such as FBXO39 poses inherent challenges in drug development, primarily due to their lack of typical active pockets and their functional reliance on protein-protein interaction interfaces, which makes the design of small-molecule inhibitors extremely difficult [54, 55]. Future research could explore indirect modulation strategies, such as utilizing PROTAC technology to degrade key downstream substrates of FBXO39 or developing peptide/peptidomimetic molecules that disrupt the FBXO39-p53 interaction [56]. Furthermore, targeting downstream nodes of this pathway (e.g., LDHA inhibitors) or combining them with existing chemotherapeutic agents may represent more feasible therapeutic approaches.

**Fig. 8:**
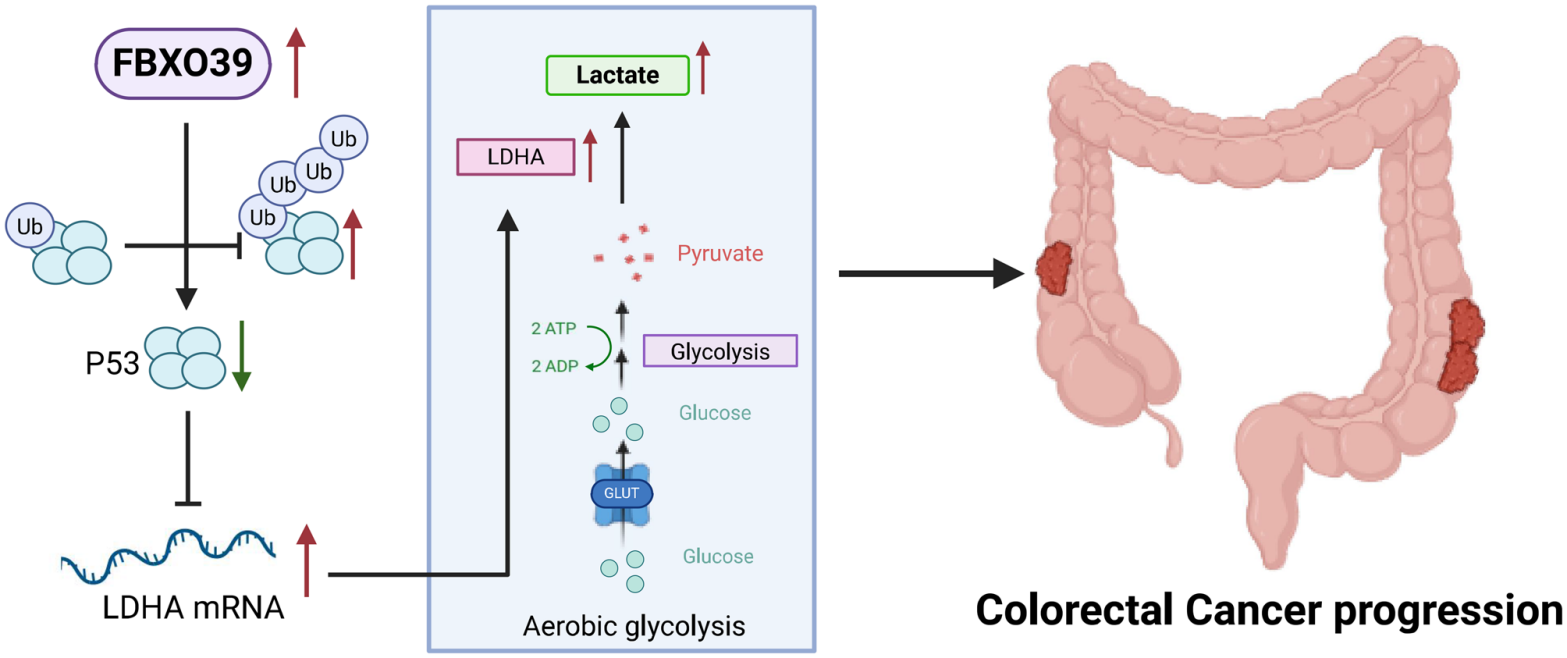
A model summarizing the role of FBXO39 in CRC.FBXO39 promotes the ubiquitin-mediated degradation of p53 protein, thereby enhancing LDHA-driven aerobic glycolysis to drive CRC progression

We therefore hypothesized that FBXO39 promotes LDHA-mediated aerobic glycolysis and tumor cell proliferation by ubiquitinating and degrading p53 protein in CRC cells, thereby relieving p53-mediated transcriptional suppression of LDHA. Experimental validation demonstrated that FBXO39 knockdown suppresses LDHA expression, while FBXO39 overexpression upregulates LDHA; restoring LDHA expression in FBXO39-knockdown cells reverses the proliferation decrease caused by FBXO39 knockdown; FBXO39 knockdown leads to p53 upregulation, LDHA downregulation, and inhibition of cell proliferation and aerobic glycolysis, while concurrent p53 knockdown restores these phenotypes. Direct mechanistic studies, including ChIP-qPCR and luciferase reporter assays, confirmed that p53 binds to the LDHA promoter and represses its transcription, and this repression is alleviated by FBXO39. Moreover, FBXO39 knockdown shows no significant effect on LDHA expression in p53-deficient HCT116 cells. In summary, we confirmed that FBXO39 promotes CRC progression by facilitating p53 ubiquitination and degradation, thereby relieving p53-mediated transcriptional repression of LDHA and upregulating LDHA-mediated aerobic glycolysis (Fig. 8).

## Electronic supplementary material

Below is the link to the electronic supplementary material.

Supplementary Material 1

## Data Availability

The datasets used during the current study are available from the corresponding author on reasonable request.

## Abbreviations

2-DG: 2-Deoxyglucose
ATP: Adenosine Triphosphate
CCK-8: Cell Counting Kit-8
CHX: Cycloheximide
Co-IP: Co-Immunoprecipitation
CRC: Colorectal Cancer
ECAR: Extracellular Acidification Rate
EdU: 5-Ethynyl-2’-deoxyuridine
FBXO: F-BOX
FCCP: Carbonyl cyanide-p-trifluoromethoxyphenylhydrazone
G6P: Glucose-6-phosphate
HR: Hazard Ratio
IHC: Immunohistochemistry
LDHA: Lactate Dehydrogenase A
OCR: Oxygen Consumption Rate
OS: Overall Survival
DFS: Disease-Free Survival
qRT-PCR: Quantitative Real-Time Polymerase Chain Reaction
Rote/AA: Rotenone/Antimycin A
TCGA: The Cancer Genome Atlas

## References

[1] Sung H, Ferlay J, Siegel RL, Laversanne M, Soerjomataram I, Jemal A, et al. Global cancer statistics, 2020: GLOBOCAN estimates of incidence and mortality worldwide for 36 Cancers in 185 countries. CA Cancer J Clin. 2021;71:209–49. 33538338 10.3322/caac.21660

[2] Dekker E, Tanis PJ, Vleugels JLA, Kasi PM, Wallace MB. Colorectal cancer. Lancet. 2019;394:1467–80. 31631858 10.1016/S0140-6736(19)32319-0

[3] Biller LH, Schrag D. Diagnosis and treatment of metastatic colorectal cancer: a review. JAMA. 2021;325:669–85. 33591350 10.1001/jama.2021.0106

[4] Shin AE, Giancotti FG, Rustgi AK. Metastatic colorectal cancer: mechanisms and emerging therapeutics. Trends Pharmacol Sci. 2023;44:222–36. 36828759 10.1016/j.tips.2023.01.003 PMC10365888

[5] Zhong X, He X, Wang Y, Hu Z, Huang H, Zhao S, et al. Warburg effect in colorectal cancer: the emerging roles in tumor microenvironment and therapeutic implications. J Hematol Oncol. 2022;15:160. 36319992 10.1186/s13045-022-01358-5 PMC9628128

[6] Offermans K, Jenniskens JCA, Simons C, Samarska I, Fazzi GE, van der Meer JRM, et al. Association between mutational subgroups, Warburg-subtypes, and survival in patients with colorectal cancer. Cancer Med. 2023;12:1137–56. 35785488 10.1002/cam4.4968 PMC9883416

[7] Nicolini A, Ferrari P. Involvement of tumor immune microenvironment metabolic reprogramming in colorectal cancer progression, immune escape, and response to immunotherapy. Front. Immunol. 2024;15:1353787. 39119332 10.3389/fimmu.2024.1353787 PMC11306065

[8] Pang B, Wu H. Metabolic reprogramming in colorectal cancer: a review of aerobic glycolysis and its therapeutic implications for targeted treatment strategies. Cell Death Discov. 2025;11:321. 40659604 10.1038/s41420-025-02623-5 PMC12259946

[9] Janic A, Valente LJ, Wakefield MJ, Di Stefano L, Milla L, Wilcox S, et al. DNA repair processes are critical mediators of p53-dependent tumor suppression. Nat Med. 2018;24:947–53. 29892060 10.1038/s41591-018-0043-5

[10] Tornesello ML. TP53 mutations in cancer: molecular features and therapeutic opportunities (review). Int J Mol Med. 2025;55. 10.3892/ijmm.2024.5448 PMC11554381 39450536

[11] Li W, Li L, Yang H, Shi C, Lei Z, Guo L, et al. Unraveling the role of TP53 in colorectal cancer therapy: from wild-type regulation to mutant. Front Biosci (Landmark Ed). 2024;29:272. 39082342 10.31083/j.fbl2907272

[12] Kennedy MC, Lowe SW. Mutant p53: it’s not all one and the same. Cell Death Differ. 2022;29:983–87. 35361963 10.1038/s41418-022-00989-y PMC9090915

[13] Yan S, Zhan F, He Y, Zhu Y. Ma Z: p53 in colorectal cancer: from a master player to a privileged therapy target. J Transl Med. 2025;23:684. 40537809 10.1186/s12967-025-06566-4 PMC12178040

[14] Huang Y, Jiao Z, Fu Y, Hou Y, Sun J, Hu F, et al. An overview of the functions of p53 and drugs acting either on wild- or mutant-type p53. Eur J Med Chem. 2024;265:116121. 38194777 10.1016/j.ejmech.2024.116121

[15] Wang Z, Liu P, Inuzuka H, Wei W. Roles of F-box proteins in cancer. Nat Rev Cancer. 2014;14:233–47. 24658274 10.1038/nrc3700 PMC4306233

[16] Ciechanover A. The unravelling of the ubiquitin system. Nat Rev Mol Cell Biol. 2015;16:322–24. 25907614 10.1038/nrm3982

[17] Cheng J, Liu O, Bin X, Tang Z. F-box proteins in cancer: from cancer cells to the tumor microenvironment. Cell Commun Signal. 2025;23:433. 10.1186/s12964-025-02445-z PMC12519858 41084060

[18] Kipreos ET, Pagano M. The F-box protein family. Genome Biol. 2000;1: Reviews 3002. 10.1186/gb-2000-1-5-reviews3002 PMC138887 11178263

[19] FeiFei W, HongHai X, YongRong Y, PingXiang W, JianHua W, XiaoHui Z, et al. FBX8 degrades GSTP1 through ubiquitination to suppress colorectal cancer progression. Cell Death Dis. 2019;10:351. 31024008 10.1038/s41419-019-1588-z PMC6484082

[20] Yuan X, Zhang Z, Jiang K, Wang X, Li Y. Preliminary study of the role F-Box protein 32 (FBXO32) in colorectal neoplasms through the transforming growth factor beta (TGF-β)/Smad4 signalling pathway. Med Sci Monit. 2018;24:1080–88. 10.12659/MSM.908030 PMC5829536 29465067

[21] Nie H, Xu H, Yang S, Tian C, Wang T, Jin C, et al. FBXO44 regulates FOXP1 degradation through AURKA-Dependent phosphorylation to promote colorectal cancer progression. Adv Sci (Weinh). 2025;e15458. 10.1002/advs.202415458 PMC12713037 41051444

[22] Ji J, Jing A, Ding Y, Ma X, Qian Q, Geng T, et al. FBXO5-mediated RNF183 degradation prevents endoplasmic reticulum stress-induced apoptosis and promotes colon cancer progression. Cell Death Dis. 2024;15:33. 38212299 10.1038/s41419-024-06421-2 PMC10784456

[23] Ge MK, Zhang N, Xia L, Zhang C, Dong SS, Li ZM, et al. FBXO22 degrades nuclear PTEN to promote tumorigenesis. Nat Commun. 2020;11:1720. 32249768 10.1038/s41467-020-15578-1 PMC7136256

[24] Yang Y, Zhao Y, Sun G, Zuo S, Chai J, Xu T, et al. FBXO39 predicts poor prognosis and correlates with tumor progression in cervical squamous cell carcinoma. Pathol Res Pract. 2022;238:154090. 36049441 10.1016/j.prp.2022.154090

[25] Zheng J, You W, Zheng C, Wan P, Chen J, Jiang X, et al. Knockdown of FBXO39 inhibits proliferation and promotes apoptosis of human osteosarcoma U-2OS cells. Oncol Lett. 2018;16:1849–54. 30008875 10.3892/ol.2018.8876 PMC6036412

[26] Koo KY, Moon K, Song HS, Lee MS. Metabolic regulation by p53: implications for cancer therapy. Mol Cells. 2025;48:100198. 39986611 10.1016/j.mocell.2025.100198 PMC11925517

[27] Stine ZE, Schug ZT, Salvino JM, Dang CV. Targeting cancer metabolism in the era of precision oncology. Nat Rev Drug Discov. 2022;21:141–62. 34862480 10.1038/s41573-021-00339-6 PMC8641543

[28] Li Q, Qin Y, Wei P, Lian P, Li Y, Xu Y, et al. Gas1 inhibits Metastatic and metabolic phenotypes in colorectal carcinoma. Mol Cancer Res. 2016;14:830–40. 27401611 10.1158/1541-7786.MCR-16-0032

[29] Wang M, Zhou Q, Cao T, Li F, Li X, Zhang M, et al. Lactate dehydrogenase A: a potential new target for tumor drug resistance intervention. J Transl Med. 2025;23:713. 40598234 10.1186/s12967-025-06773-z PMC12210585

[30] Sharma D, Singh M, Rani R. Role of LDH in tumor glycolysis: regulation of LDHA by small molecules for cancer therapeutics. Semin Cancer Biol. 2022;87:184–95. 36371026 10.1016/j.semcancer.2022.11.007

[31] Xia T, Meng L, Xu G, Sun H, Chen H. TRIM33 promotes glycolysis through regulating P53 K48-linked ubiquitination to promote esophageal squamous cell carcinoma growth. Cell Death Dis. 2024;15:740. 39389957 10.1038/s41419-024-07137-z PMC11467421

[32] Keum N, Giovannucci E. Global burden of colorectal cancer: emerging trends, risk factors and prevention strategies. Nat Rev Gastroenterol Hepatol. 2019;16:713–32. 31455888 10.1038/s41575-019-0189-8

[33] Ciardiello F, Ciardiello D, Martini G, Napolitano S, Tabernero J, Cervantes A. Clinical management of metastatic colorectal cancer in the era of precision medicine. CA Cancer J Clin. 2022;72:372–401. 35472088 10.3322/caac.21728

[34] Breuer E, Hebeisen M, Schneider MA, Roth L, Pauli C, Frischer-Ordu K, et al. Site of recurrence and survival after Surgery for colorectal peritoneal metastasis. J Natl Cancer Inst. 2021;113:1027–35. 33484560 10.1093/jnci/djab001

[35] Zheng L, Shen J, Chen Y, Lin J, Li P, Zhao X, Ren H, Sun Y, Wang Z, et al. FBXO43 promotes cell cycle progression in cancer cells through stabilizing SKP2. Cancer Lett. 2024;591:216848. 10.1016/j.canlet.2024.216848 38604312

[36] Tekcham DS, Chen D, Liu Y, Ling T, Zhang Y, Chen H, et al. F-box proteins and cancer: an update from functional and regulatory mechanism to therapeutic clinical prospects. Theranostics. 2020;10:4150–67. 32226545 10.7150/thno.42735 PMC7086354

[37] Wu J, Yao F, Li Y, Zhao Z, Liu J, Xu T, et al. The cancer-testis antigen FBXO39 predicts poor prognosis and is associated with stemness and aggressiveness in glioma. Pathol Res Pract. 2022;239:154168. 36244247 10.1016/j.prp.2022.154168

[38] Liu YC, Yan S, Liu DM, Pei DX, Li YW. Aberrant expression of cancer-testis antigen FBXO39 in breast cancer and its clinical significance. Clin Lab. 2020;66. 10.7754/Clin.Lab.2020.200121 32902238

[39] Song MH, Ha JC, Lee SM, Park YM, Lee SY. Identification of BCP-20 (FBXO39) as a cancer/testis antigen from colon cancer patients by SEREX. Biochem Biophys Res Commun. 2011;408:195–201. 21338577 10.1016/j.bbrc.2011.02.077

[40] Huang K, Han Y, Chen Y, Shen H, Zeng S, Cai C. Tumor metabolic regulators: key drivers of metabolic reprogramming and the promising targets in cancer therapy. Mol Cancer. 2025;24:7. 39789606 10.1186/s12943-024-02205-6 PMC11716519

[41] Qin R, Fan X, Huang Y, Chen S, Ding R, Yao Y, et al. Role of glucose metabolic reprogramming in colorectal cancer progression and drug resistance. Transl Oncol. 2024;50:102156. 39405607 10.1016/j.tranon.2024.102156 PMC11736406

[42] Chen H, Gao S, Liu W, Wong CC, Wu J, Wu J, et al. RNA N(6)-methyladenosine methyltransferase METTL3 facilitates colorectal cancer by activating the m(6)A-GLUT1-mTORC1 axis and is a therapeutic target. Gastroenterology. 2021;160:1284–300.e1216. 33217448 10.1053/j.gastro.2020.11.013

[43] Liu H, Chen X, Wang P, Chen M, Deng C, Qian X, Bai J, Li Z, Yu X, et al. PRMT1-mediated PGK1 arginine methylation promotes colorectal cancer glycolysis and tumorigenesis. Cell Death Dis. 2024;15:170. 10.1038/s41419-024-06544-6 PMC10894231 38402202

[44] Zhao L, Yu N, Zhai Y, Yang Y, Wang Y, Yang Y, et al. The ubiquitin-like protein UBTD1 promotes colorectal cancer progression by stabilizing c-myc to upregulate glycolysis. Cell Death Dis. 2024;15:502. 39003255 10.1038/s41419-024-06890-5 PMC11246417

[45] Ni X, Lu CP, Xu GQ, Ma JJ. Transcriptional regulation and post-translational modifications in the glycolytic pathway for targeted cancer therapy. Acta Pharmacol Sin. 2024;45:1533–55. 10.1038/s41401-024-01264-1 PMC11272797 38622288

[46] Liao M, Yao D, Wu L, Luo C, Wang Z, Zhang J, et al. Targeting the Warburg effect: a revisited perspective from molecular mechanisms to traditional and innovative therapeutic strategies in cancer. Acta Pharm Sin B. 2024;14:953–1008. 38487001 10.1016/j.apsb.2023.12.003 PMC10935242

[47] Qi Y, Tan Z, Chen H, Xiao Z, Zhang L, Wu B, et al. Pyrimidine synthase CAD deamidates and inactivates p53. Cell Res. 2025;35:520–23. 40240485 10.1038/s41422-025-01112-9 PMC12205080

[48] Zong Z, Xie F, Wang S, Wu X, Zhang Z, Yang B, Zhou F, et al. Alanyl-tRNA synthetase, AARS1, is a lactate sensor and lactyltransferase that lactylates p53 and contributes to tumorigenesis. Cell. 2024;187:2375–92.e 2333. 10.1016/j.cell.2024.04.002 38653238

[49] Liebl MC, Hofmann TG. The role of p53 signaling in colorectal cancer. Cancers (Basel). 2021;13. 10.3390/cancers13092125 PMC8125348 33924934

[50] Li H, Sun L, Gao P, Hu H. Lactylation in cancer: current understanding and challenges. Cancer Cell. 2024;42:1803–07. 39393355 10.1016/j.ccell.2024.09.006

[51] Jing Z, Liu Q, He X, Jia Z, Xu Z, Yang B, Liu P, et al. NCAPD3 enhances Warburg effect through c-myc and E2F1 and promotes the occurrence and progression of colorectal cancer. J Exp Clin Cancer Res. 2022;41:198. 10.1186/s13046-022-02412-3 PMC9188166 35689245

[52] Zhang Q, Qin Y, Sun X, Bian Z, Liu L, Liu H, Mao L, Sun S, et al. Sodium butyrate blocks the growth of colorectal cancer by inhibiting the aerobic glycolysis mediated by SIRT4/HIF-1α. Chem Biol Interact. 2024;403:111227. 10.1016/j.cbi.2024.111227 39241941

[53] Zhou Y, Niu W, Luo Y, Li H, Xie Y, Wang H, et al. p53/Lactate dehydrogenase a axis negatively regulates aerobic glycolysis and tumor progression in breast cancer expressing wild-type p53. Cancer Sci. 2019;110:939–49. 30618169 10.1111/cas.13928 PMC6398928

[54] Li Y, Bao K, Sun J, Ge R, Zhang Q, Zhang B, et al. Design of PROTACs utilizing the E3 ligase GID4 for targeted protein degradation. Nat Struct Mol Biol. 2025;32:1825–37. 40295770 10.1038/s41594-025-01537-1

[55] Ebadi P, Stratton CM, Olsen SK. E3 ubiquitin ligases in signaling, disease, and therapeutics. Trends Biochem Sci. 2025;50:960–76. 40940201 10.1016/j.tibs.2025.07.009 PMC12435903

[56] Zhang J, Chen C, Chen X, Liao K, Li F, Song X, Liu C, Su MY, Sun H, Hou T, et al. Linker-free PROTACs efficiently induce the degradation of oncoproteins. Nat Commun. 2025;16:4794. 10.1038/s41467-025-60107-7 PMC12102262 40410168

