# Supplementary material for: FBXO39 promotes LDHA-mediated aerobic glycolysis and colorectal cancer progression by p53 degradation
Source: J Transl Med. 2026 Apr 1;24:547. doi: 10.1186/s12967-026-08056-7 (PMC13093999; doi:10.1186/s12967-026-08056-7)

Figure 2

Fig 2C up

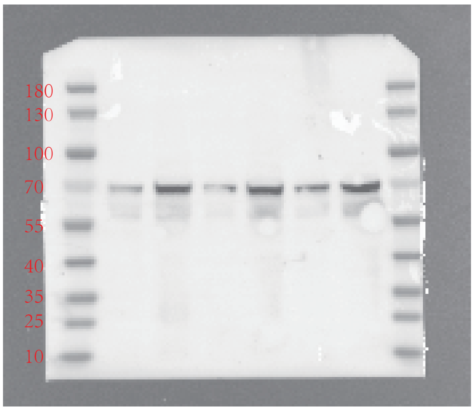

FBX039

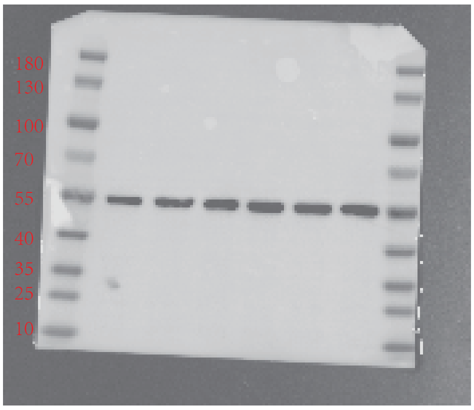

tubulin

Fig 2C down

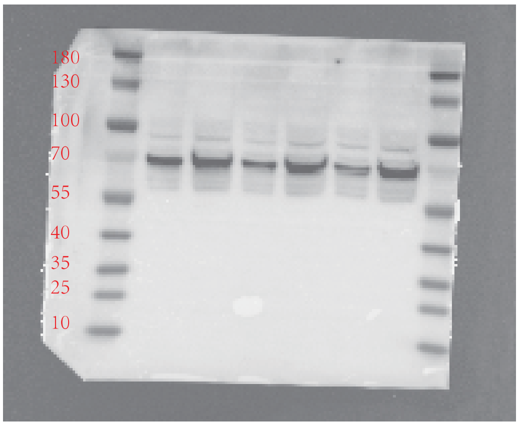

FBX039

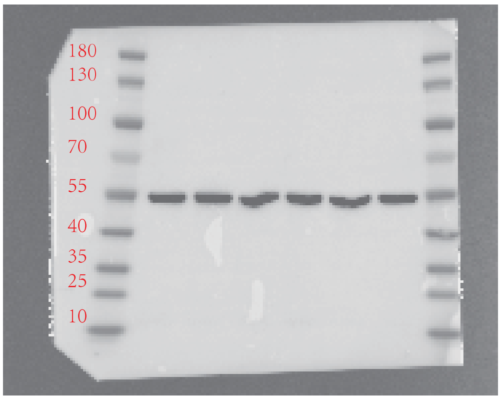

tubulin

Figure 3

Fig 3A

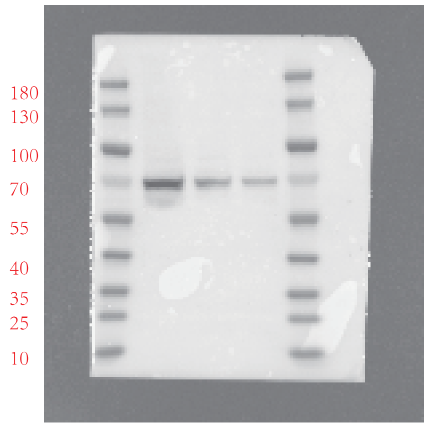

FBX039

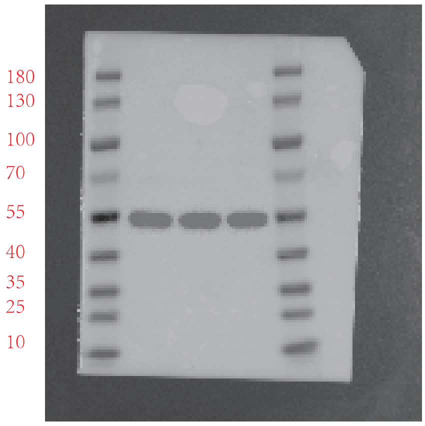

tubulin

Fig 3B

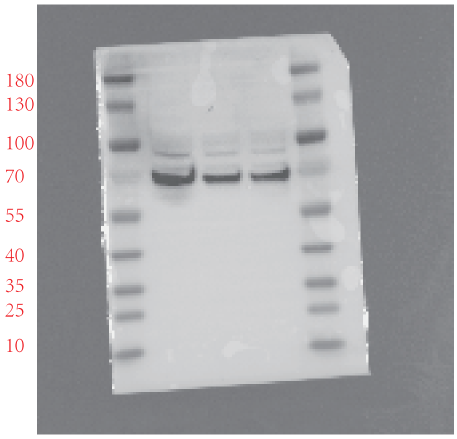

FBX039

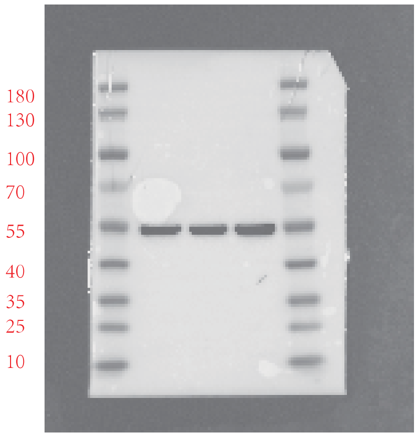

tubulin

Fig 3C

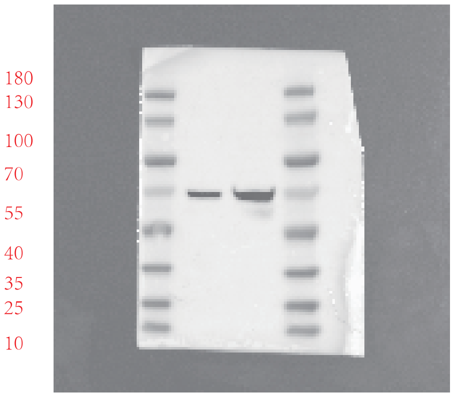

FBX039

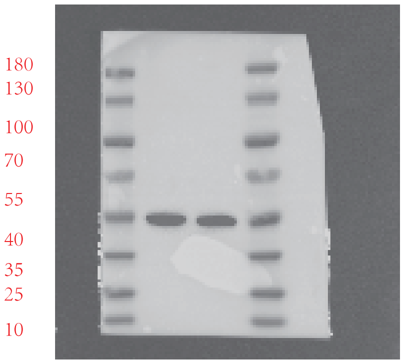

tubulin

Fig 3D

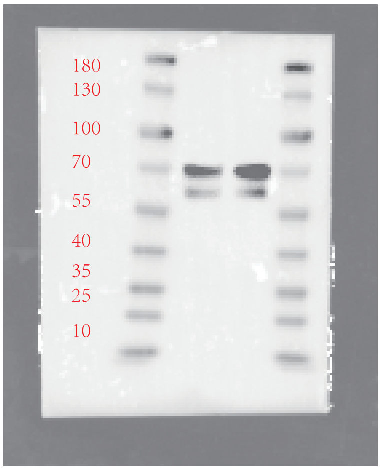

FBX039

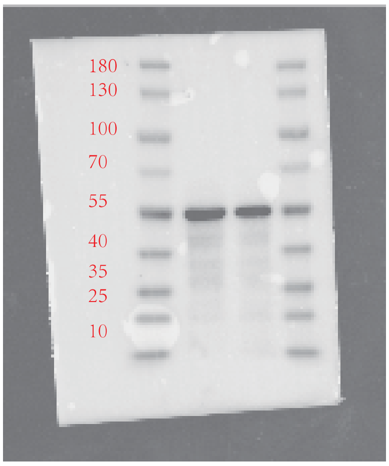

tubulin

Figure 4

Fig 4A

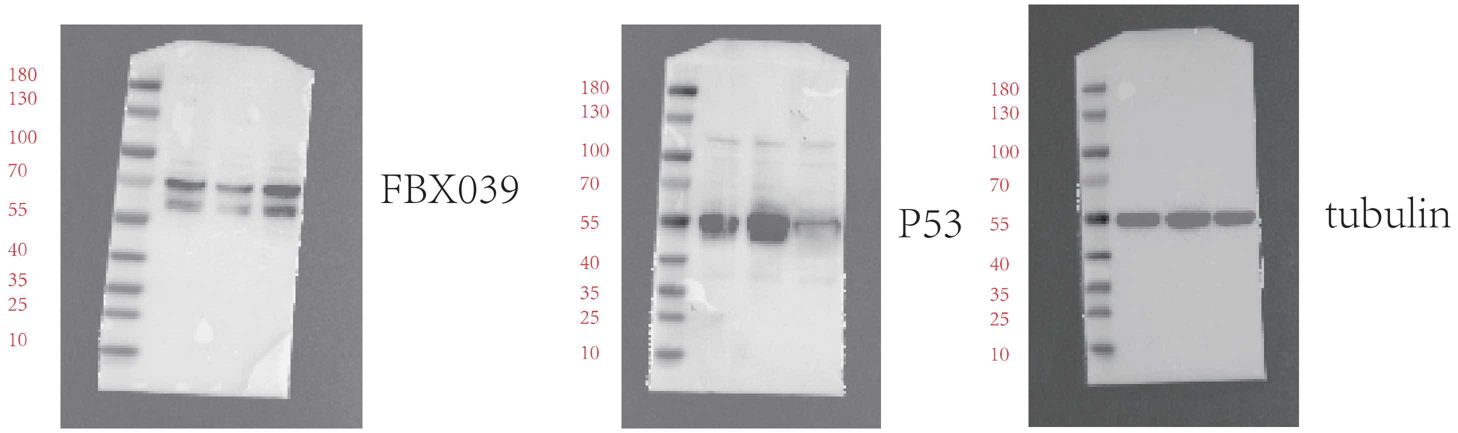

Fig 4B

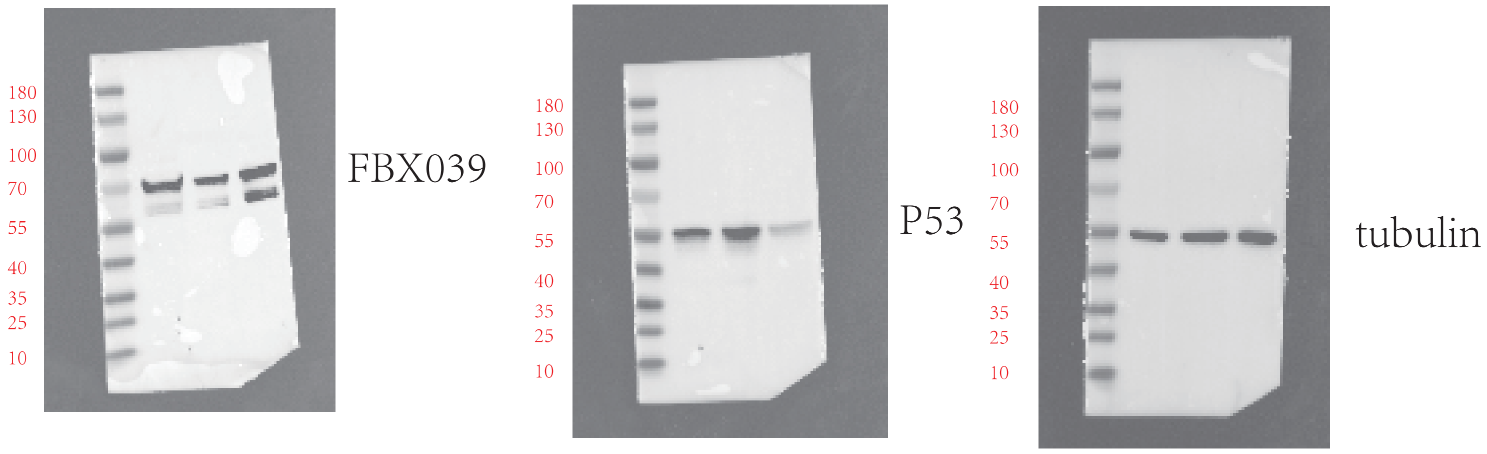

Fig 4G

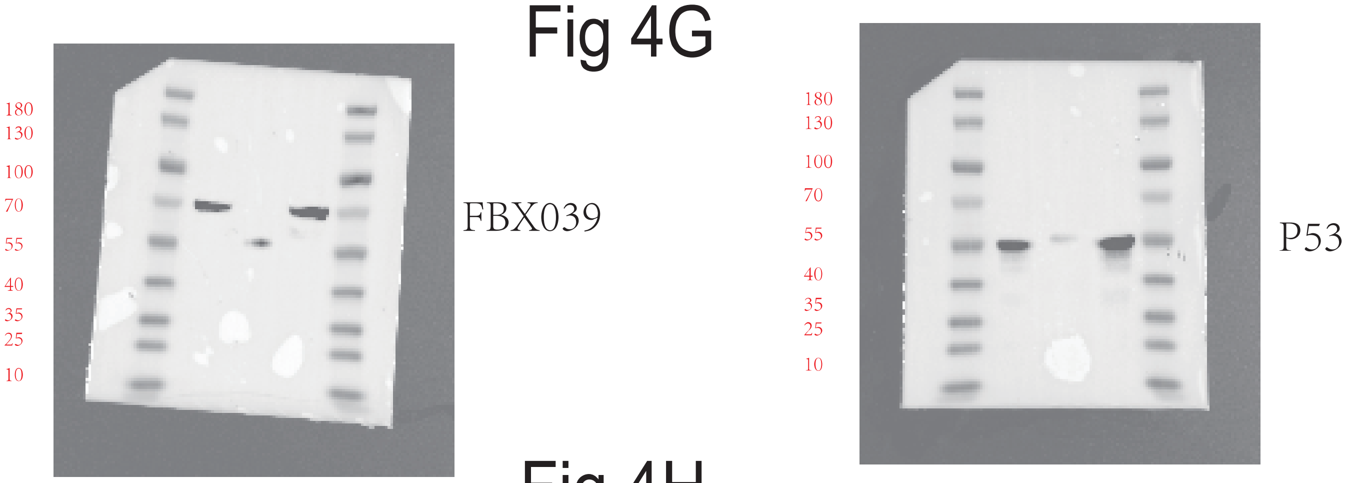

Fig 4H

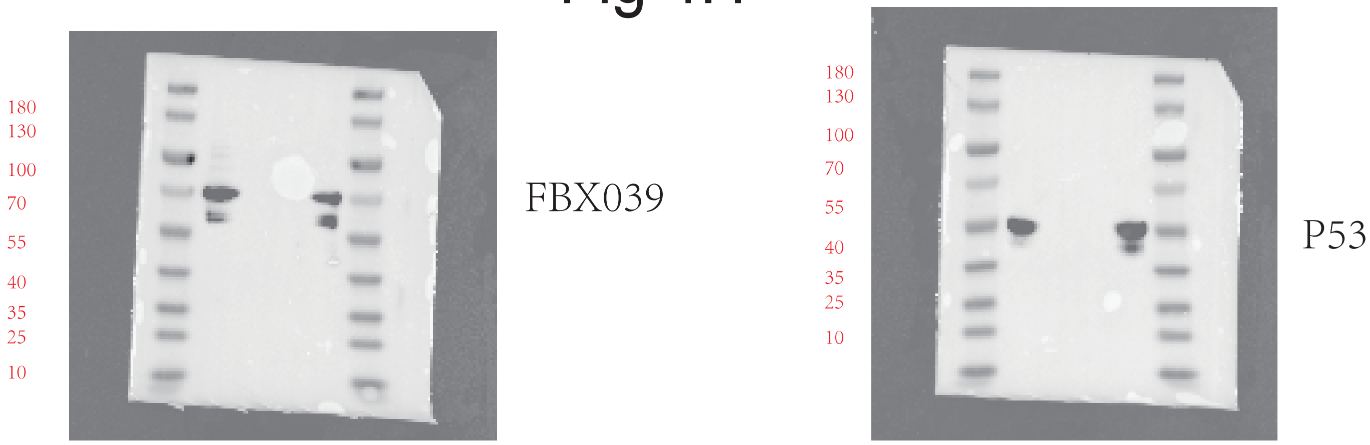

Figure 5

Fig 5A

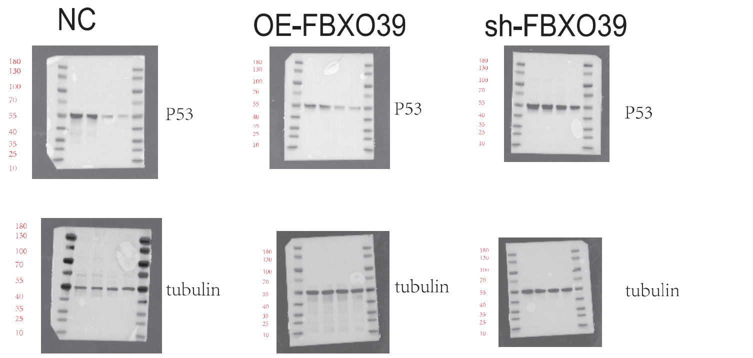

Fig 5C

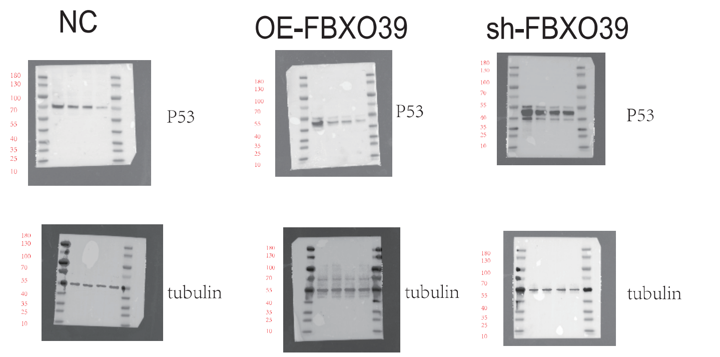

Fig 5E

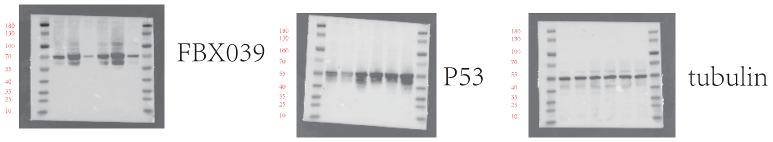

Fig 5F

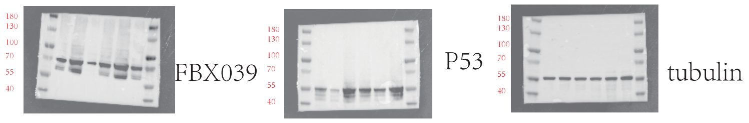

Fig 5G

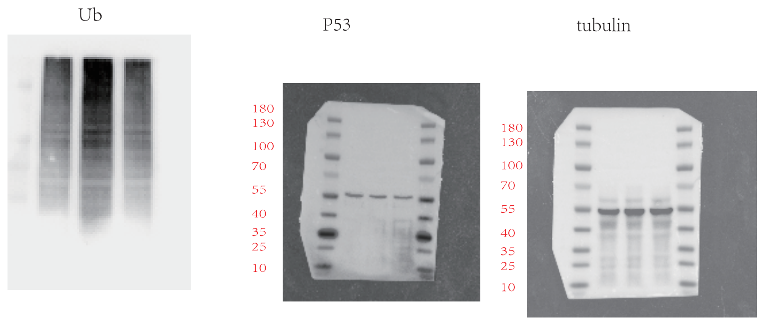

Fig 5H

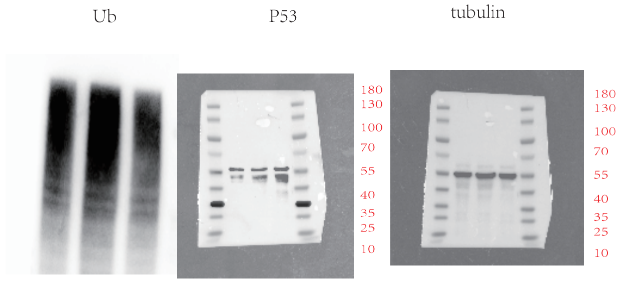

Fig 5I

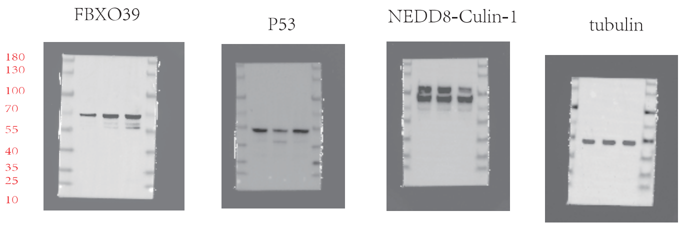

Fig 5J

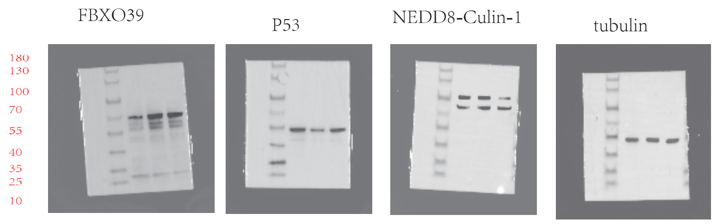

Figure 7

Fig 7A Left RKO

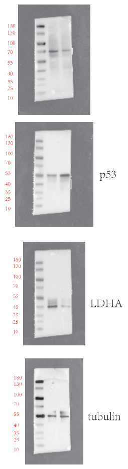

Fig 7A right HCT116

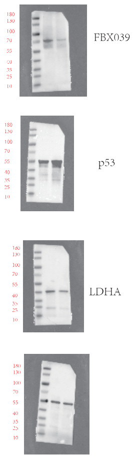

Fig 7C Left RKO

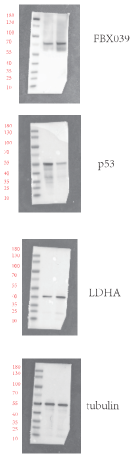

Fig 7C right HCT116

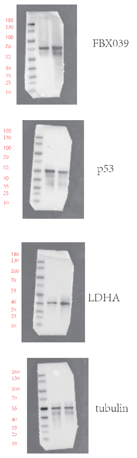

Fig 7I

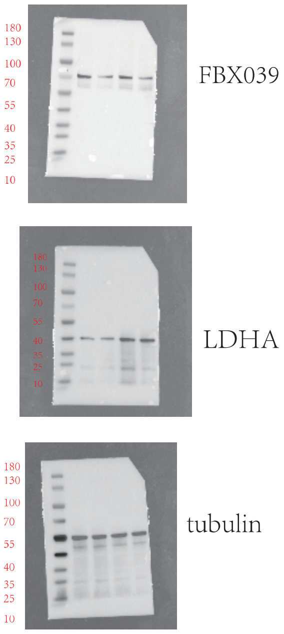

Fig 7K`

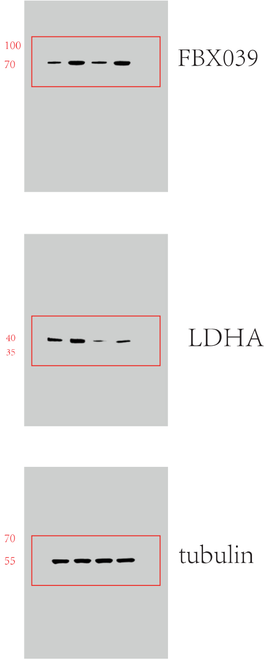

Fig 7O

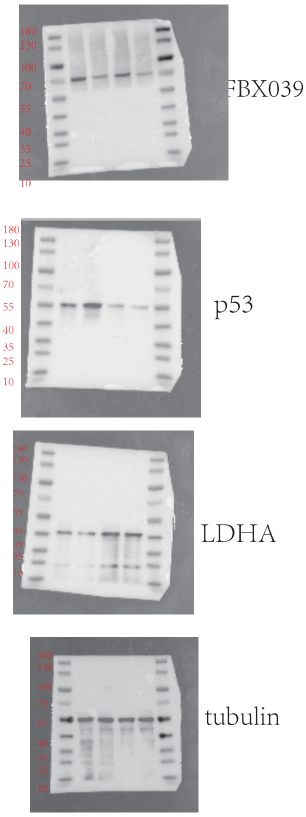

Fig 7T

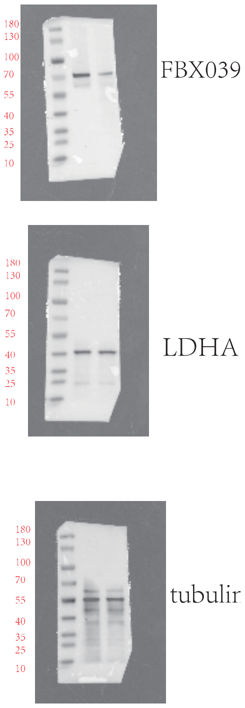

Fig 7V

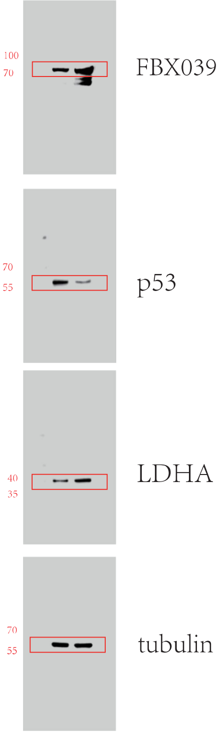

Supplement: Supplementary file 1 — Supplementary Material 1 [file 12967_2026_8056_MOESM1_ESM.pdf]
